# Investigating the Sources of Silver in 17th- and 18th-Century Silver Coins from the *Rooswijk* Shipwreck by Compositional Studies

**DOI:** 10.3390/ma18050925

**Published:** 2025-02-20

**Authors:** Francesca Gherardi, Jan Pelsdonk

**Affiliations:** 1 Investigative Science, Historic England, Fort Cumberland, Portsmouth PO4 9LD, UK; 2 Rijksmuseum, 1071 XX Amsterdam, The Netherlands; duit@live.nl; 3 Teylers Museum, 2011 CH Haarlem, The Netherlands

**Keywords:** silver coins, trace elemental analysis, shipwreck, µXRF, SEM-EDS, VOC

## Abstract

The colonisation of the Americas and the discovery of its rich ores had a great impact on the world economies, making them quickly become the main suppliers of precious metals in Europe. The compositional studies of several coins (ducatons, eight reales cob8, four reales cob4, eight reales pillar dollar, four reales half pillar dollars, rijderschellings and silver rijders) recovered from the 18th-century Dutch East India Company *Rooswijk* wreck by micro X-ray fluorescence (µXRF) spectroscopy revealed further knowledge about the silver trade and the silver sources used to produce coins in mints in the Low Countries over a wide timeframe (1618–1739). The results provided trace elemental ‘fingerprints’ of coins minted with silver from known mines, and matching against them revealed the silver sources used in coins, whose mint location could not be identified due to their poor state of preservation. This study proved that, despite the decrease in silver production in European mines in the 17th century and the huge influx of American silver into Europe, in the 18th century, the mints in the Dutch Republic and, to a lesser extent, in the Spanish Netherlands still highly relied on the recycling of older coins and on the import of silver from central European mines.

## 1. Introduction

The *Rooswijk* was a Dutch East India Company (Verenigde Oostindische Compagnie, VOC, 1602–1799) vessel built in 1737 in the Republic of the Seven United Netherlands (further referred to as the Dutch Republic) [1]. It sank in 1740 on the Goodwin Sands (about 6 miles off the Deal coast in Kent, UK; National grid reference TR4951658901) during its second journey from Texel (Netherlands) to Batavia (Jakarta, Indonesia). The vessel was a ‘retourship’, which is a type of VOC vessel designed for long voyages of 18 months to three years, typically from Europe to Batavia.

The ship carried a wide range of personal objects and trade goods, including coins, ingots, sabre blades, glass beads, and small and large copper alloy and pewter objects. It is a rare instance of the survival of a VOC retourship in North European waters and with a cargo of goods to be exported to Indonesia from Europe [2].

The shipwreck was discovered by divers in 2004 and was subsequently protected under the Protection of Wrecks Act (1973). The vessel and its cargo are owned by the Dutch Government and are managed as a protected wreck site by Historic England (on behalf of the UK Department of Digital, Culture, Media and Sport). During the excavations of 2017 and 2018, funded by the Cultural Heritage Agency of the Netherlands (RCE) (on behalf of the Dutch Ministry of Education, Science and Culture) and managed in the UK by MSDS Marine in the framework of the #Rooswijk1740 project, many artefacts were recovered and transported to Historic England, Fort Cumberland laboratories (Portsmouth, UK) for their conservation and post-excavation studies [3,4,5]. The coins are currently stored at the National Maritime Depot at Batavialand in Lelystad, The Netherlands. 

### 1.1. Aims

**Figure 1:**
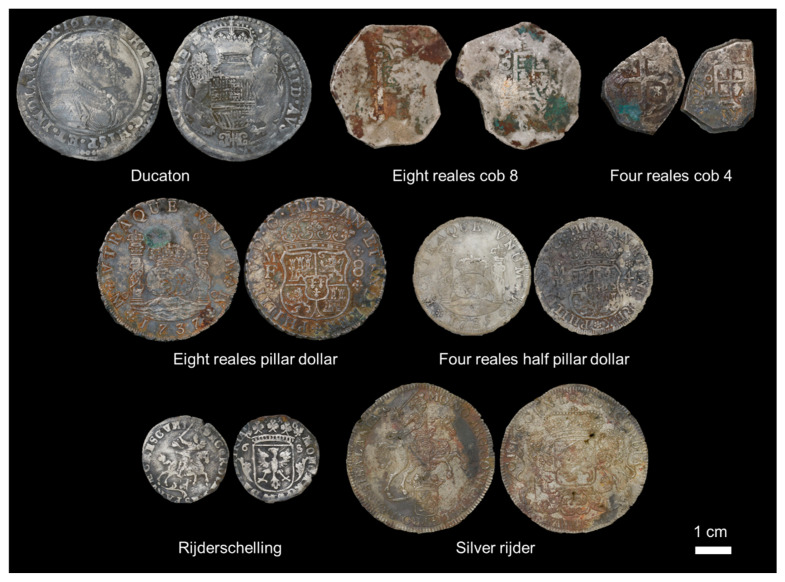
Typologies of coins recovered from the *Rooswijk*: ducaton, eight reales cob8, four reales cob4, eight reales pillar dollar, four reales half pillar dollar, rijderschelling (or six stuiver) and silver rijder ©Rooswijk1740 project.

During the excavations of 2017 and 2018, thousands of coins were found. These can mainly be divided into four groups: Spanish-American reales next to silver rijders and rijderschellings from the Dutch Republic and ducatons from the Spanish Netherlands (Figure 1). Although a lot of these coins were produced shortly before the *Rooswijk* started its journey, much older coins were also found; the oldest ones were produced in 1618 and were well over 100 years old when the ship sank. A small historical background is provided to place these coins into their time period.

The aim of this research is to study the elemental composition of a selection of coins recovered from the *Rooswijk* shipwreck to identify the trace elemental fingerprints of coins of known typology, date, mint, sovereign issuer and assayers to be used as reference for coins which could not be identified due to their poor state of preservation.

Micro X-ray fluorescence (µXRF) spectroscopy was used to study the coins as it is minimally invasive, and it allows rapid qualitative and quantitative multielement analysis without sampling. Since it is a surface technique, special attention should be paid if the thickness of the corrosion layers on the coins exceeds the depth of the X-radiation.

Compositional studies were carried out to gain data about silver suppliers of mints in the Spanish Netherlands and in the Dutch Republic, which are not associated with a specific mine, highlighting changes in sources, silver recycling and the re-melting of old coins over time. The impact of the import of silver from the Americas to Europe and its trade in Asia was evaluated to answer questions about long-distance exchange routes.

Finally, the study aimed to gain further knowledge about the monetary system in the Netherlands, regarding the use of old and Spanish-American coins and the purity of silver in different coin typologies.

### 1.2. World Silver Movement

The VOC had intense intra-Asian trading networks around Batavia and was established to gain a monopoly on specific Asian goods [6,7]. It soon became the largest European commercial and colonial power in the far East.

Many of the products imported into Europe were luxury goods such as silk, porcelain, diamonds, pearls, ivory and wooden furniture. Other Eastern goods like cotton from the Philippines, Indian linen, spices, tea and coffee were also traded for the European market [6,7]. The different commercial, diplomatic and colonial relationships resulted in various forms of cultural exchange, cross-cultural connections and influences.

In return, the high demand for precious metals in Asian countries led to the intense export of silver and (to a lesser extent) gold from Europe to Asia for Eastern goods [7,8,9,10]. Because of this lucrative trade, the VOC shipped large amounts of silver ingots and coins to Asia to purchase Eastern goods in the period June 1739–May 1740, for a total of 3.8 million guilders for the VOC-chambers Amsterdam and Zeeland [11]. It was also quite common that crew members of VOC vessels would bring additional silver coins to Asia for (illegal) private trade or to profit from the different exchange rates.

The high silver content of the large coins made them useful for international trade. They were far less in use in domestic circulation, where other coin types prevailed [12]. However, the transported coins were not only used for trading. Some types—copper duiten and coins with a low silver content (one and two stuivers and rijderschellings with a value of six stuiver)—were also employed as local currency by the Dutch themselves in Batavia and in other locations under VOC control. Their payment value was much higher than the value of the metal used to produce the coins [12]. During the 17th century, the minting of small coin denominations for use in the Dutch Republic itself was restricted because of the huge amount of circulating coins of bad quality. Only the VOC obtained permission from the government to order small coins if they promised to export them without exception to Asia [12,13]. The production of rijderschellings was not restricted, but problems existed in Dutch circulation, since not all of them reached the mandatory amount of silver. Therefore, in 1694, the rijderschellings of good silver were stamped with a countermark. All non-countermarked rijderschellings were reduced in payment value to 5.5 instead of 6 stuiver. Because of this, the lower-valued coins were more likely to be transported abroad (where they could still be used for their face value of 6 stuiver), as the pieces in the *Rooswijk* clearly show; none of them are countermarked.

Coins were never simply minted with a random amount of silver. The issuing authority determined the silver content very precisely. Mint masters were only allowed to deviate from this by a fraction. The mandatory silver compositions of the main types of coins in the *Rooswijk* were 944/1000 (ducatons), 941/1000 (silver rijders), 930/1000 (cobs), 917/1000 (pillar dollars), and 583/1000 (rijderschellings) [14].

Silver coins and ingots were the main cargo in VOC ships on outbound trips, and this explains the recovery of high amounts of silver coins and bullions from wreck sites associated with these trading vessels directed to Asia.

### 1.3. Silver Sources

Until the beginning of the 16th century, most of the silver circulating in Europe was extracted from mines in central Europe, in particular from Bohemia, Saxony, Slovakia, Hungary, and Tyrol [15,16]. New finds of silver ores in the 1480s led to the production of the first big silver coins (talers). In the 15th and 16th centuries, high amounts of silver from central Europe were reaching Antwerp, which quickly became an important commercial centre [8].

Starting from the middle of the first quarter of the 17th century, the silver production in all the European mining centres decreased [9,15]. This was not only caused by the exhaustion of mines. The European colonisation of the Americas had an enormous impact on the world economy, as with the discovery of rich silver ores in South and Central America, the new silver reached Europe in huge quantities [16,17]. The European mining industry received a blow, because the silver extraction in Europe was more expensive than in Spanish America. In other words, Spanish-American silver was ‘cheaper’. The European mines kept on producing at a lower rate, with less profit, and the precious metals used in Europe were mainly imported from the American colonies. In the seventeenth and eighteenth centuries, 85–90% of the world’s silver came from the Americas [9].

Spain controlled these mines, the transport of silver (and gold) in Spanish ports and the trade to other European countries [18]. A total of circa 86,000 tons of silver and 1700 tons of gold was mined in the American colonies up to the 1800s, representing about 70–80% of the world stock of silver and 40% of the world’s gold stock throughout the 18th century [19].

The main silver mines were based in Potosí (Viceroyalty of Peru, in present-day Bolivia) and in Mexico (Viceroyalty of New Spain). Minor mines in Peru, Bolivia, Chile and Guatemala were active as well [9,16,17,19]. In Peru and Mexico, the silver production was concentrated in a few mines within few small regions, and many of them were highly exploited over short periods of several decades [9]. It was, however, quite challenging to provide a timeline of the production of each mine due to incomplete data collected and registered to the Crown offices by the mine officers and owners.

In addition to Europe, the silver produced in the Americas was traded to the Philippines with an increase in exports in the eighteenth century [9].

In Asia, especially in the sixteenth and seventeenth centuries, several silver mines were exploited in Japan with a maximum production of about 150–200 tons per year in the early 17th century, which declined by the 1640s and stayed quite low thereafter [7,9,16,19]. The silver extracted in Japan was exported not only to Europe but also to East and Southeast Asia (especially China) [9].

Spanish-American silver coins were a trusted commodity in Europe in the 17–19th centuries due to their size, finesse and weight, although a big scandal with secretly debased coins in Potosí in the second quarter of the 17th century led to an international trade crisis [20,21]. Spanish-American coins reached the Netherlands (Spanish Netherlands and Dutch Republic) in huge quantities, mainly to be used in trade, to make general silverware or to be sent to local mints as raw material to produce local coins [7].

Spanish-American silver was distributed to Spain and from there sold to European creditors and transported in huge quantities to many major cities and ports in southern and western Europe, particularly, during the 17th century, in Amsterdam and Middleburg in the Netherlands towards Amsterdam [22,23]. Despite trade embargoes during the Eighty Years’ War (1568–1648) between Spain and the Dutch Republic, high amounts of Spanish-American silver reached Amsterdam, for instance, through other trade centres (Hamburg, Barcelona and Genova, or through France and England) [8,10].

The Spanish-American coins and ingots were re-melted in the European mints to produce new coins. Thanks to the huge influx of new silver, in the 16th century, the still-circulating medieval European coins were re-melted and replaced by new, big silver coins with a higher concentration of silver [24]. The recycling of older coins was a common practice, which was carried out in organised and controlled systems by skilled workers. Initially, the silver used for these new coins contained silver from European sources. Although the influx of European silver did not stop abruptly, from the mid-16th century, more and more silver originated from Spanish-America, which resulted in coins with different proportions of old and new silver.

### 1.4. Silver Extraction and Refining Techniques

In European sources, silver was mainly extracted from cerussite (PbCO_3_) and mostly galena (PbS) by smelting and subsequent cupellation [25]. Smelting was the first stage of the process, which is based on roasting the metal ore (for sulphide ores) to oxidise it, then heating the metal ore with a reducing agent (charcoal). Cupellation was a purification stage aimed at separating metals which oxidise more easily (such as copper and lead), from silver, which remains metallic [26].

This process was used for recycling metal objects with a large copper content. In the 15th century, the Saiger process was introduced in the German silver mines to more easily separate silver from copper ores using lead in smelting and achieve high metal yields [27,28]. This technological development spread in different regions in central Europe and led to an increase in the silver production in European mines [27,28].

In the Americas, the silver ore in Potosí was discovered by the Europeans around 1545, but a large quantity of silver was produced only after the introduction of the mercury amalgamation technique (also called the Patio process) in the 1550s in Mexico and in the 1570s in Peru [29,30]. Silver was mainly extracted from silver sulphide compounds, which could be produced by weathering surface layers rich in silver chlorides and other halides, together with elemental silver (in Andean deposits) and argentiferous galena (in Mexican deposits) [31]. From the second half of the sixteenth century, the exhaustion of the easily accessible and high-grade silver ores resulted in a decrease in the silver yields obtained from American sources [30,31]. Smelting was still conducted for argentiferous galena ores, but the Saiger process was not sustainable in the American mines, as considerable amounts of timber, which were not available there, were required to produce charcoal. The Patio process allowed silver recovery from low-grade mines and to process large volumes in a simpler way, leading to the rapid development of silver production in the Americas [30,31]. In this process, the silver ore was crushed with brine and mixed with mercury, and then copper sulphate was added, and the mix was spread in shaded areas. After the formation of the amalgam, silver was extracted by heating, during which process the mercury evaporated. Mercury came from a few sources: Huancavelica in Peru, Almadén in Spain, and Idria in Slovenia. The Spanish detained the monopoly of mercury, which was distributed in the Americas [9,31,32]. The Patio process was particularly efficient for ores with low amounts of or no sphalerite, iron and copper ores and low contents of arsenic and antimony [26].

Further technological developments introduced in the Patio process allowed scaling up the amalgamation of silver ore at the industrial level. In this way, the American mines could be exploited intensively and produce copious quantities of silver to be transported to mainland Spain.

### 1.5. Compositional Studies of Silver Coins and Artefacts and the Determination of Silver Provenance 

The study of the composition of the alloys in silver objects can help in the interpretation of the manufacturing technology and the silver sources for the identification of cupellation, recycling and mixing of different silver batches.

The presence of trace elements in silver artefacts can be influenced by many factors: the geographical origin of the ore, the method of refining used, recycling and the specific recipe chosen by the metal worker in the production of the object.

There are several challenges when studying the provenance of metal archaeological artefacts using trace elements to establish trade routes [33,34]. First, the chemical composition of the same ore is not homogenous, which makes it challenging to link it to a metal artefact.

Secondly, the extraction and refining techniques affect the composition of the original ore, as additional elements, such as mercury, with the Patio process, and copper in silver alloys, can be introduced. Alloying with copper and copper alloys can affect the trace elemental composition of the silver ore, as they can bring additional trace and minor elements. In addition, the high temperature required in smelting ores can lead to changes in the concentration of the trace and minor elements [35]. In this way it is difficult to determine whether and to what extent these processes have modified the trace elements in the composition of a metal artefact.

Further-on, metal re-melting and recycling induce changes in the trace element composition of the original ores. However, it has been proved that silver from a mint, which uses silver from different sources, still displays a distinctive trace elemental fingerprint that is unique to the specific mint [36]. This observation enables the evaluation of silver sources at different times and its geographic circulation.

Finally, archaeological artefact corrosion during burial, especially in the marine environment, affects the surface elemental composition due to elemental leaching and surface enrichment. A wide range of corrosion products can be found, from silver chlorides and sulphides to copper oxides and chlorides [37]. In addition, because of the presence of iron objects on the wreck site and because artefacts are often found encased in concretions, silver objects are often covered by a red-brown iron oxide patina. Corrosion often occurs in specific areas and not homogenously (in terms of thickness and composition) over the whole object, resulting in changes in the elemental composition of different areas of the same artefact.

Gold (Au) and bismuth (Bi) have been identified as potential discriminators of silver ores in the study of silver artefacts, as they are not significantly affected by metallurgical processes, and they are consistent with the original mine [33,38,39,40,41,42]. Bismuth mostly oxidises in the last stages of the cupellation, and it is present in very small proportions in the produced silver [42,43]. The bismuth/silver (Bi/Ag) and bismuth/lead (Bi/Pb) ratios can be used to differentiate different silver suppliers and production sites, which is especially useful in the investigation of coins, where the elemental fingerprint of the silver ores is modified during re-melting and recycling [40,41,44,45].

Several studies on silver artefacts and coins minted in Europe (mainly Spain and Germany) and in the Americas in the 15th–19th century were successful in the evaluation of the alloy composition and state of preservation [46,47,48] and in determining the silver sources by studying the composition of trace elements by X-ray fluorescence (XRF) spectroscopy [49,50], energy-dispersive X-ray spectroscopy (EDS), particle-induced X-ray emission (PIXE), proton and neutron activation analysis [29,34,35,51,52,53,54,55,56], and inductively coupled plasma mass spectrometric analysis (ICP-MS) [24,29,57,58].

These studies proved that the differences in the concentration of gold, indium and tin in coins minted in South America and Mexico can give information about the location of specific silver sources [29,51,52]. For instance, the variation in the gold and bismuth content provided insight into the provenance of silver sources used in Portuguese coins minted from the 15th to the 17th century [41].

Gentelli analysed several silver coins minted in the Americas and in Europe and artefacts from the Western Australian Maritime Museum’s collection, which were recovered from different wreck sites [57,58,59,60]. In the study, the trace elemental fingerprint of coins produced in different mints in the Americas and in Europe was established, allowing the researchers to use inter-elemental ratios as a tool to date and evaluate the provenance of silver archaeological objects and coins.

The results obtained from this research were compared to the elemental analysis of Dutch coins and medals in the collection of the Teylers Museum (Haarlem, the Netherlands), allowing the separation between Spanish American coins minted in Potosí and in Mexico [24].

Other studies on silver coins integrated silver, copper and lead isotopes ratios, which are influenced by the geological conditions and the time formation of the ores, to track the silver circulation in the 16th–18th century [24,61].

Most of the coins analysed in the present study were minted using different silver batches, which resulted in a trace elemental composition different from the original silver ores. The coins minted in the Americas (Mexico and Potosí) were probably produced with silver from a single, local mine [36,57,58,59]. The results reported here confirm those of previous studies, which proved that silver from a mint, which uses different silver sources and is not connected to a single local source, still shows a unique trace elemental composition [25,27,36,59].

## 2. Materials and Methods

### 2.1. The Coins of the Assemblage

A total of 275 coins recovered from the *Rooswijk* were selected and analysed in this research (Table S1). All the selected coins were visually identified in terms of typology, and in some cases, it was possible to observe the mint, the date, the sovereign issuer and the assayer. The selection aimed at providing a representation of the wide range of mints and dates for the different coin typologies to compare different silver suppliers.

The group comprises ducatons (46 coins minted in the Spanish Netherlands in Antwerp, Bruges, Brussels and Tournai, from 1618 to 1704), eight reales cob8 (46 coins minted in Mexico and Bolivia, Potosí, from 1724 to 1735), four reales cob4 (37 coins minted in Mexico, from 1724 to 1735), eight reales pillar dollars (42 coins minted in Mexico, from 1734 to 1738), four reales half pillar dollars (2 coins minted in Mexico, from 1735 to 1736), rijderschellings (or six stuivers) (54 coins minted in the Dutch Republic in Deventer, Groningen, Harderwijk, Kampen, Nijmegen, Utrecht, Zutphen and Zwolle from 1673 to 1691) and silver rijders (48 coins minted in the Dutch Republic in Deventer, Dordrecht, Harderwijk, Hoorn, Kampen and Utrecht, from 1652 to 1739).

To produce coins, sheets of silver were normally brought to the desired thickness by hammering or using a roller press, after which coin flans were cut out. The cob coins were produced differently; cut from silver bars, into chunks with a defined weight but irregular shapes. Finally, the coin flans were stamped with dies—either hammered or with the use of a screw press—to add the designs on both sides. In those days, the value of a coin was determined for a significant part by the amount of silver used. Automatically, the more valuable coins, like ducatons, pillar dollars, silver rijders and cobs, became thicker than coins of low value, like the rijderschellings. The first were very thick (about 4 mm), while the rijderschellings were much thinner (about 1 mm).

### 2.2. Methods

Most of the coins were found encased in dense concretions formed on the seabed around corroding iron objects. The coins were analysed after being removed from the concretion and after desalination in deionised water.

To study the microstructure and evaluate corrosion and any changes in the chemical composition between the surface and the bulk, 11 coins, partially broken or showing defects, were sampled, embedded in resin, ground and polished to a 0.25 µm finish. Polishing was challenging due to the small samples and the soft nature of the metal in combination with hard, mineral inclusions that sometimes detached from any remaining surface concretions.

The samples were analysed by scanning electron microscopy (SEM) using an FEI-Inspect F (FEI, Hillsboro, OR, USA) combined with an energy dispersive spectrometer (EDS) INCA X-Act (Oxford Instruments, High Wycombe, UK) (SEM-EDS). The samples were coated with 15 nm of carbon, and the images were collected using a back-scattered electron (BSE) detector. SEM was used because it allows the study of the samples at higher magnifications than optical microscopy. The EDS data were collected from at least three representative areas of the surface (within the first 150 µm) and the bulk of the samples at 25 KeV and quantified using the Oxford Instruments INCA software. The aim was to investigate any silver surface enrichment or copper leaching in the coins. The analysed areas had the same size, and the analyses were carried out at the same magnifications for all the coins.

Prior to the analysis of the 275 selected coins, a small area from the edge of the coin least affected by corrosion was gently scratched with a scalpel under the microscope to attempt to expose the bare metal, which was analysed by μXRF, using a Bruker M4 Tornado μXRF spectrometer (Billerica, MA, USA), with a rhodium tube and a 30 mm^2^ silicon drift detector. The data were collected in point mode (point size of 20 µm) at 50 kV and 400 µA with a vacuum. An AlTi 100/25 sandwich filter was used to minimise diffraction peaks. The spectra were deconvoluted using the software provided, and Kα lines were used to quantify all the elements but Au, Hg, Pb and Bi, for which Lα lines were selected as their Kα lines are out of range.

The machine was calibrated using certified reference materials (silver, gold and copper alloys standards) of known composition to cover a wide range of elements (Table S2). The tabulated results are averages of at least three analyses and normalised. Because the analysed areas were not always flat and uniform, a reduction of the collected signal occurs, which can impact the results.

The reference materials were also used to quantify the limits of detection (LODs) of the minor elements: LOD below 100 ppm for Ni, Zn and Bi; LOD below 150 ppm for Sn, Pb and Fe; LOD below 200 ppm for As and Sb; and LOD below 550 ppm for Au. The detection limit for Au is overestimated due to the lack of available reference materials with Au concentrations 10 times below the presumed detection limit. The LOD for Au can be estimated using elements with similar absorption effects in silver-based alloys to be about 100–150 ppm. A similar estimation was considered for mercury.

The accuracy of the μXRF results of the silver and gold alloys was evaluated with the analysis of certified reference materials, and the relative error was calculated as ((certified content − obtained content)/certified content) × 100). The relative error was lower than 3.5% for major and minor elements, proving the good accuracy of the method.

Starting from the normalised μXRF data (Table S3), principal component analysis (PCA) was applied to four minor elements (Ni, Zn, Au and Bi) using R software (4.4.2 version). PCA was performed by using the built-in R function princomp(), the data were centered and scaled before the analysis, and the coordinates on each principal component were calculated. All values below the detection limit were replaced with zero and included in the PCA calculations. PCA is a statistical technique for reducing and simplifying the dimensionality of large datasets, minimising information loss and increasing the interpretability of the dataset [62]. It considers the largest variances in multiple element concentrations between analyses to identify new variables (the principal components) that are linear functions of those in the original dataset, the variance, and are uncorrelated with each other. The principal components can be used to evaluate coin signatures for comparison and grouping.

## 3. Results

### 3.1. Morphological and Compositional Investigations of Cross-Sections by SEM-EDS

**Figure 2:**
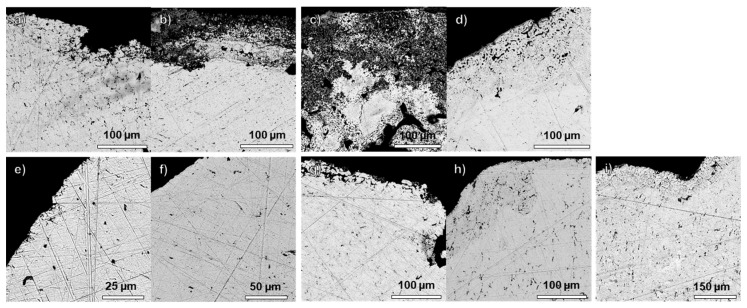
SEM images (BSE detector) of cross-sections from four reales cob4 (RK17A00689 and RK17A00422, (**a**) and (**b**), respectively), eight reales cob8 (RK17A00361 and RK17A00333, (**c**,**d**)), ducatons (RK17A02853 and RK17A02959, (**e**,**f**)), silver rijders (RK17A00689 and RK17A02785, (**g**,**h**)), and eight reales pillar dollar (RK17A00096, (**i**)).

Except for ducatons (Figure 2e,f), which are in good condition, the sampled coins show increased porosity close to the surface (Figure 2). The thickness of the porous layer is not uniform, indicating different levels of corrosion developed in different areas of the same coin.

The thickness of this layer ranges from 100 to 150 µm in the four reales cob4 (RK17A00422) and the eight reales cob8 (RK17A00361 and RK17A00333), and it is about 25–50 µm thick in the four reales cob4 (RK17A00280), the silver rijders (RK17A00689 and RK17A02785) and the eight reales pillar dollar (RK17A00096) (Figure 2).

Some of the coins have additional iron-rich corrosion layers on top of the porous one, which are probably formed inside the concretion (Figure S1b–d).

No intergranular corrosion is visible in the bulk of the coins, except in cob8 (RK17A00361), which is more brittle and shows cracks and voids, probably caused by trapped oxygen within the metal (Figure 2c). The presence of cracks has most probably exacerbated the corrosion of this coin.

In the bulk of the coins, some copper-rich phases (darker grey) can be identified (Figure S1), and in some of the coins, they appear elongated and oriented parallel to the surface, which are probably the results of cold-working (i.e., hammering) [60].

The composition of the bulk and the porous surface layers of the coins was analysed to evaluate any copper leaching and silver surface enrichment on the coin surface, which could result in misleading surface µXRF results. Indeed, in the case of corroded artefacts, the composition of the surface will vary compared to the bulk. In excavated coins made of silver and copper-based alloys, copper tends to leach from the surface, resulting in silver surface enrichment.

The results prove that the chemical composition of the surface and the bulk of most of the coins is very similar, with minor changes in the silver and copper content (Table 1).

The copper content is higher on the surface than the bulk in the very brittle and corroded eight reales cob8 coin (RK17A00361), probably due to increased copper leaching from the bulk and redeposition on the surface.

These results support prior findings that when coins have a silver content higher than 91 wt%, hardly any silver enrichment occurs and no significant differences in the chemical compositions (in terms of main elements silver, copper and lead) of the bulk and the porous surface layers of the coins can be observed. This is consistent with other studies in the literature carried out on coins with a silver content higher than 91.2% [60,63,64,65,66], which is the maximum value of solid copper solubility in silver [67]. Although the surface is altered, the elements of interest are still present and the ratios of these elements to each other are broadly similar to in the unaltered metal. In addition, the surface alteration layers tend to be thinner, so the bulk metal is more easily accessed by preparing a small area of the surface.

This indicates that the surface analysis of corroded silver with high silver content (higher than 91 wt%) is representative of the bulk, unless severe corrosion and brittleness are visible, as in the case of eight reales cob8 (RK17A00361). Coins with an alloy less rich in silver need further investigation to identify the thickness of the corrosion layers, and attention should be paid to select the best area to carry out compositional analysis to be representative of the bulk composition.

**Figure 3:**
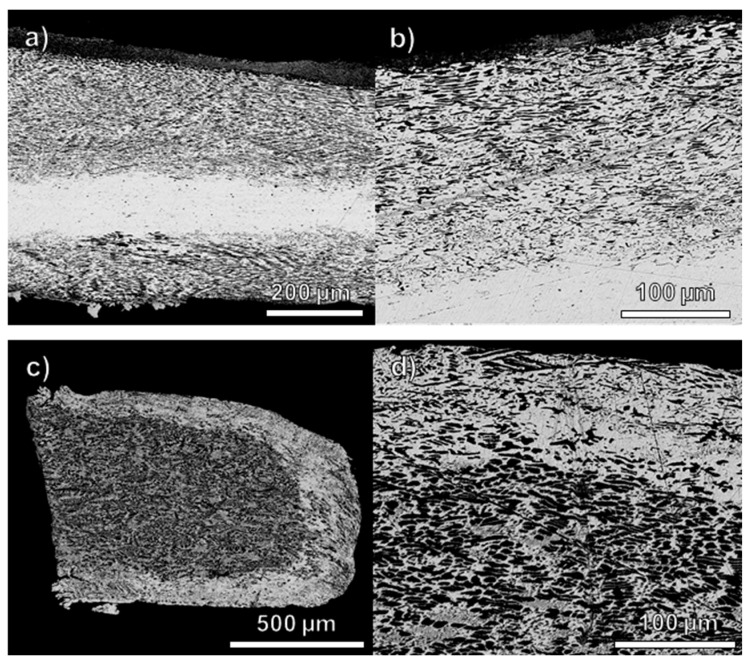
SEM images (BSE detector) at different magnifications of cross-sections from rijderschellings (RK17A00842 (**a**,**b**) and RK17A00877 (**c**,**d**)).

The rijderschellings (RK17A00842 and RK17A00877) are smaller and thinner coins that exhibit thicker and more porous surface layers (about 200 µm thick) compared to the other coins (Figure 3). One coin (RK17A00842) displays a structure with a silver-rich bulk (brighter area) and elongated copper-rich phases on the top and bottom, closer to the surface (Figure 3a,b). The other coin (RK17A00877) is characterised by an increased number of copper-rich phases in the bulk, which run parallel to the surface (Figure 3c,d and Figure S1c).

One rijderschelling (RK17A00877) presents a bulk composition different from the rest of the coins, as it contains about 81 wt% Ag and 18 wt% Cu. The porous surface layer from this coin is richer in silver (about 92 wt%) than the bulk, indicating that surface enrichment occurs in this case. This result was not found in the other investigated rijderschelling, which was minted using an alloy richer in silver (about 92 wt%), indicating a possible use of different types of alloys and the different manufacture for this type of coins.

The mandatory silver content of these coins was 583/1000 [14], but the measured silver content is higher than 80 wt%, highlighting that the investigation of the microstructure of a higher number of rijderschellings is required to fully understand the type of alloy and manufacturing procedure used for their production.

### 3.2. Compositional Study of the Coins by μXRF

The coins were analysed by μXRF to obtain a trace elemental ‘fingerprint’ of different types of coins, which were minted in various locations at different times (Table S1).

The compositional data highlight that ducatons, eight reales cob8, four reales cob4, four reales half pillar dollars, eight reales pillar dollars and silver rijders contain high concentrations of silver (higher than 91 wt%) and up to about 8 wt% of copper, which was added to increase the strength and hardness of silver (Table S3 and Figure S3a).

The rijderschellings are smaller and thinner coins, which are richer in copper (up to about 28 wt%). The issuing authorities determined that the silver content of these coins was 583/1000 [14]. However, although they are heavily corroded in many cases, the measured silver content in these coins is higher than 90 wt% for 80% of the coins, between 80 and 89 wt% for 13% of the coins and between 70 and 79 wt% for 7% of the coins (Table S3 and Figure S3a). As highlighted in the results below, the increased copper content of rijderschellings than the other typologies results in increased lead concentrations in some of the coins. However, the higher copper and lead concentrations are not directly correlated with the increased content of some trace elements (zinc and nickel), which are associated with copper used as an alloying agent. Their trace elemental composition is similar to some coins of other typologies and with a lower copper content.

This means that only a higher heterogeneity and a negligible difference in the composition of trace elements occur in coins with slightly higher copper content. Compared to the coins with a high percentage of silver, thicker corrosion layers were found in the schellings, formed by copper that had leached out and resulting in silver enrichment. This indeed is the case, but even after the removal of the surface layer under a microscope, the analysis shows that the composition of the bulk is quite consistent with the surface. The question remains why the measured silver content in the bulk is so much higher than the mandatory silver content. After all, these coins do not have a countermark (see Section 1.2) and are likely to contain less than 583/1000 silver.

The coins display low contents of minor and trace elements (Ni, Zn, Au, Hg, Pb and Bi), which were not deliberately added. These elements are naturally occurring and associated with silver ores, alloying, refining and recycling [26,33]. The single content of Ni, Zn, Au, and Bi does not exceed 0.5 wt%, while the Hg and Pb concentrations are below 1 and 1.5 wt%, respectively (Table S3 and Figure S2).

During the cupellation process, impurities in argentiferous lead (antimony, arsenic, tin, iron, zinc, mercury, nickel and to a lesser extent copper and bismuth) are removed, and between 0.1 and 1% lead is left in silver [26,33,39,68]. The gold/silver ratio does not change significantly in metallurgical processes, and bismuth mostly oxidises in the last phases of cupellation, making them good discriminators of silver sources [33,38,39,40,41,42].

About 34% of the analysed coins contain a Pb content lower than 0.1 wt%, which could indicate that non-cupelled silver was used (Table S3 and Figure S3a). This group mainly comprises coins minted in the Americas and some ducatons, as expected, because the Patio process was mainly used to refine silver in the Americas. The rest of the coins (rijderschellings, silver rijders and some ducatons) were probably manufactured using cupelled silver sources.

The Au content varies in the different coin typologies, being higher in coins minted in Mexico (eight reales cob8, four reales cob4, four reales half pillar dollars and eight reales pillar dollars), which is most probably related to local silver ores richer in gold (Table S3, Figures S2 and S3b). The gold content has a wide range within coins minted in the same location, as Au/Ag ratio can range over two or more orders of magnitude within a single deposit [69].

The bismuth concentration in coins minted in Spanish America varies, indicating different levels of this element in the silver source used in their manufacturing (Table S3, Figures S2 and S3b). The Bi content is particularly high in the silver rijders minted in Kampen (Overijssel) in 1739, which could be linked to a different refining process or ore type (see Section 3.2.3).

The concentration of mercury is slightly higher in coins minted in Spanish America, apart from some rijderschellings, which contain up to 0.8 wt% of mercury (Table S3 and Figure S2). The presence of mercury in the coins from Spanish America is probably associated with residues linked to the amalgamation process for silver extraction [41]; for the rijderschellings, it could be also due to surface chemical treatment based on the mercury-silvering process.

The coins minted in the Spanish and United Netherlands (ducatons, rijderschellings and silver rijders) contain more zinc than the coins produced in Spanish America (Table S3, Figures S2 and S3b). These coins are believed to have been produced using different cupelled silver batches and recycling older coins, as their mints are not linked to a specific local silver source. During recycling, silver can be refined via cupellation or debased adding pure copper or copper alloy objects. Zinc was particularly high in some of the coins, but it should not have survived cupellation. This can be due to the used production technology (alloying with copper or copper alloys) or ore type (for example, zinc, similarly to other volatile elements, can be present in high concentrations in native silver [40]).

Nickel is present in low concentrations in the studied coins. The only exceptions are two eight reales cob8 coins and, to a lesser extent, some rijderschellings (Table S3 and Figure S2). The cob8 coin with the highest Ni content was minted in Potosí, while the mint of the other cob8 was not identified. Similarly to Zn, Ni should not survive cupellation, as PbO oxidises metallic nickel [26]. It is reported that nickel concentrates in rich ‘dry’ silver ores consisting of native silver, as well as silver sulphide and silver chloride by smelting [70]. This is common in Andean deposits [31]. Nickel is also a trace element in copper from central European ores, which could have been added in the recycling and alloying phases [71,72,73,74].

Additional elements such as tin and antimony were sought, as they can be detected by µXRF and were found in high concentrations in coins minted in Potosí in a previous study [57], but they were not detected in the analysed coins. In this context, four minor elements (Ni, Zn, Au and Bi) were used to conduct PCA.

PCA was applied to the whole dataset and to subgroups to display the multivariate data with two variables (or principle component, PC) and allow clustering. In the following paragraphs, different PCA plots are reported in each figure, and each graph shows the distribution of the coins based on typology (top left), date (top right), and location of the mint (bottom left). In addition, a graph with the variables (trace elements) represented as vectors is also included (bottom right).

#### 3.2.1. The Coins of the Assemblage

**Figure 4:**
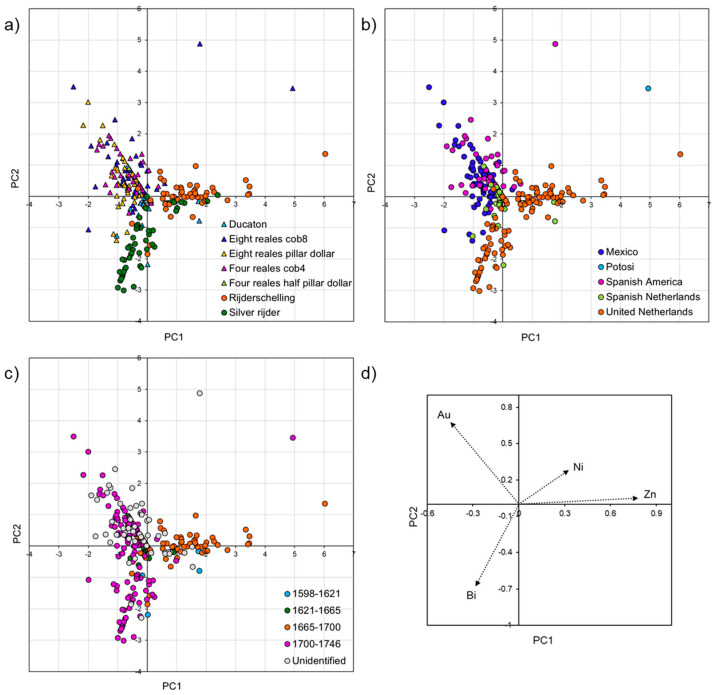
Principal component analysis (PCA) plots of the trace elemental (nickel—Ni; zinc—Zn; gold—Au; and bismuth—Bi) composition of all the coins analysed in this study. The graphs represent the data based on typology (**a**), date (**b**), location of the mint (**c**) and variables (trace elements) represented as vectors (**d**).

PCA was used to compare the trace elemental composition of the coins and better highlight their differences. The first and second components represent 31% and 29% of the variance, respectively (Figure 4).

All the coins of the assemblage have been identified in terms of typology (Figure 4a). Three main groups can be observed: the first one mainly comprises silver rijder coins, a few rijderschellings and ducatons rich in bismuth; the second one includes coins which are relatively high in gold (eight reales cob8, eight reales pillar dollars, four reales cob4, four reales half pillar dollars and most of the ducatons); and the third one mainly contains rijderschellings and some silver rijders rich in zinc. Two eight reales cob8 coins are outliers as richer in nickel.

In terms of chronology, the dataset has been divided into four groups of dates (1598–1621, 1621–1665, 1665–1700 and 1700–1746), which are linked to the Spanish sovereigns ruling in the specific timeframes (Figure 4b).

The few older coins (1598–1621, light blue symbols) are rich in bismuth and zinc, and the most recent ones (1700–1746, purple symbols) contain high concentrations of gold and bismuth.

The coins minted between 1621 and 1700 (green and orange symbols) are separated based on their relatively high zinc content; some of them contain gold and bismuth and they overlap with the most recent ones.

Finally, to evaluate the elemental fingerprint of different silver suppliers, the coins were divided based on the mint locations (Figure 4c).

The coins struck in Mexico are characterised by high gold content, which is consistent with other studies of trace elements in silver coins minted in Mexico [24,29,57,58,59].

Only one coin was identified as being minted in Potosí, Bolivia, and it contains high nickel, most probably due to the use of dry silver ores to extract silver. Previous studies on silver coins from Potosí proved that silver from this area is richer in indium [29,51,52]. Another study found that coins minted at Potosí are also relatively high in tin and antinomy [57]. However, these elements were not detected here.

Most of the coins struck in the Spanish Netherlands (ducatons) and some of the coins minted in the Dutch Republic overlap with coins minted with Mexican silver, proving the use of pure American silver to produce some of these new coins. The patterns of the coins minted in the Dutch Republic and a few of the ones minted in the Spanish Netherlands show similarities and most of them exhibit a trace elemental fingerprint which is distinctive from the coins minted in Mexico and Potosí, as they are richer in bismuth and zinc. These elements have been found in high concentrations in coins struck in central European mints (Saxony and Nuremberg) [57]. Some coins show an intermediate pattern, indicating that in these mints, different sources of silver (from both central Europe and Spanish America) have been used, and the re-melting and recycling of older coins might have contributed to the formation of a distinctive trace elemental fingerprint [36]. It is difficult to establish if the elemental signature matching central European sources is related to a supply of new silver, the recycling of older coins, or alloying with local copper and copper alloys. Similar results were also obtained in other studies on silver coins minted in the Netherlands [35,57,59].

Compared to the Dutch Republic, a much higher number of coins minted in the Spanish Netherlands used silver coming from the Americas, indicating wider circulation of American silver in this territory. An increase in the influx of American silver in the Netherlands is visible in coins struck after the peace of Westphalia (1648), which ended the Eighty Years’ War between Spain and the Netherlands and the trade embargo.

While it was challenging to match the date of unidentified coins based on the PCA plot, it was possible to overlap and predict the source of silver of some of the unidentified coins (for example the coins from Spanish America), as they showed elemental fingerprints which overlap with specific silver sources.

#### 3.2.2. The Coins Minted in the Spanish Netherlands and in Spanish America

**Figure 5:**
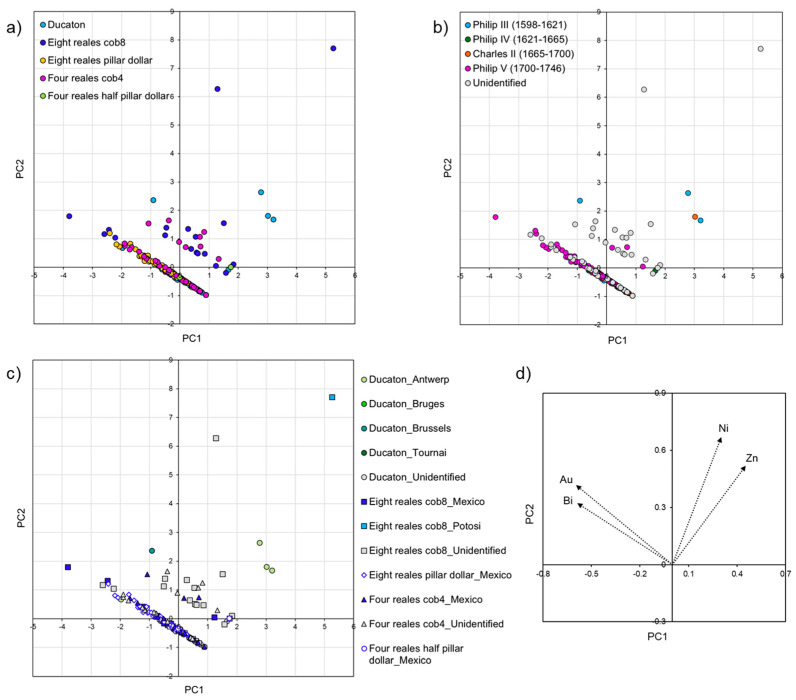
Principal component analysis (PCA) plots of the trace elemental (nickel—Ni; zinc—Zn; gold—Au; and bismuth—Bi) composition of coins from Spanish America and the Spanish Netherlands (ducatons, eight reales cob8, eight reales pillar dollars, four reales cob4 and four reales half pillar dollars) analysed in this study. The graphs represent the data based on typology (**a**), sovereign and date (**b**), location of the mint (**c**) and variables (trace elements) represented as vectors (**d**).

PCA was applied to the subgroup of coins of the assemblage minted in the Spanish Netherlands and in Spanish America to evaluate specific clusters in the coins minted in the Americas and evaluate silver influx in European mints (Figure 5). PC1 and PC2 explain 30% and 28%, respectively, of the cumulated variance.

Except for two eight reales cob8 coins, which have high nickel, the cob8, cob4 and pillar dollars form two clusters of coins rich in gold and bismuth (Figure 5a). One cluster with coins minted in Mexico and Spanish America is slightly richer in zinc and nickel than the other group, probably due to differences in the silver ores, the use of a different refining procedure (temperature and duration), or alloying with different copper sources.

Most of the ducatons overlap with this group, while others are richer in zinc.

In the assemblage, only a few coins (only ducatons) are minted during Philip III’s reign (1598–1621), and they are richer in zinc compared to coins minted later (Figure 5b). The coins of the assemblage struck under Philip IV (1621–1665) and Charles II (1665–1700) are ducatons, and almost all of them were minted using silver sources rich in gold and bismuth. The coins struck later (1700–1746) comprise cob8, cob4, pillar dollars and one ducaton. Additionally, for these coins, silver sources with high gold and bismuth contents were used to produce them.

The dataset was divided according to the different mints of production to evaluate the provenance of silver sources (Figure 5c). As previously reported, the Mexican coins are rich in gold, and several unidentified coins match this pattern [29,41,57,59].

The eight reales cob8 coin from Potosí is separate from the rest of the eight reales coins as it is richer in nickel; the elemental composition of an eight reales cob8, whose mint could not be identified (RK17A00391), overlaps with silver from Potosí.

The ducatons were minted in the Spanish Netherlands in Antwerp, Bruges, Brussels and Tournai. The coins struck in Bruges, Brussels and Tournai used pure Mexican silver sources, as well as most of the more recent coins from Antwerp. The earlier Antwerp coins are rich in zinc, similarly to coins minted in Germany [57]. For the ducatons, it is possible that the trace elemental fingerprint matching coins struck in central European mints can be the result of the use of recycled older coins produced with silver containing European copper or alloying with European copper rich in zinc. No silver from Potosí was used in these mints, as the coins do not contain nickel (associated with the coin minted in Potosí), tin or antimony, which were found in high concentrations in coins from this area [57]. This result confirms the decrease in the production of silver in the mines in Potosí after 1650 and the increase in the amount of silver mined in Mexico [16,17,29,51,75]. This is in agreement with the study by Guerra of some coins minted in the Spanish Netherlands, which were not struck with pure Potosí silver [35].

In the numismatic study (Table S1), three eight reales cob8 coins (RK17A00624, RK17A00972 and RK17A01023) stand out, since their descriptions do not match the PCA results. The mint marks and assayer’s marks are not visible. Two of these coins show a date of production in the 18th century, and the date of the third coin is not visible. Compared to all other dated coins in the *Rooswijk*, it is likely that this third coin is also produced in the 18th century. Mexico can be ruled out as their production centre, because the specific design was not in use in Mexico after 1570. This leaves Potosí and Lima, where this type was produced in the period of 1651–1739. PCA was performed on the coins from Spanish-American mints to evaluate any subgroups (Figure S4). PC1 and PC2 explain 31% and 27%, respectively, of the cumulated variance. The coins minted in Mexico divide into two quite broad clusters, one richer in zinc and nickel. The coin minted in Potosí is separate from this group due to the high level of nickel. Intriguingly, the three cob8 (yellow symbols) overlap with coins from Mexico. This result suggests that the elemental fingerprint of silver from Lima cannot always be discriminated from the Mexican one by µXRF. Further analysis by a technique able to provide additional trace elements of coins of this design minted in Lima will help fine-tune the elemental fingerprint of these coins.

Since most of the American coins were minted in Mexico, PCA was applied to this dataset to identify specific clusters (Figure S5). PC1 and PC2 represent 36% and 33% of the variance, and no specific subpopulation could be identified in terms of coin typology.

The coins were minted within a short time period, from 1724 to 1738, and no clear separation between subgroups was observed. The earlier coins seem to be slightly richer in gold and zinc, while the more recent ones are richer in bismuth, but significant overlaps occur. This is probably due to the source of silver used in Mexican mint being from local ores, which are characterised by a similar trace element composition, as found in a previous study [58]. The slight changes in the gold content in the Mexican source is expected, as the variation in Au/Ag ratio over two or more orders of magnitude can occur in the same ore [69].

#### 3.2.3. The Coins Minted in the Spanish Netherlands and in the Dutch Republic

**Figure 6:**
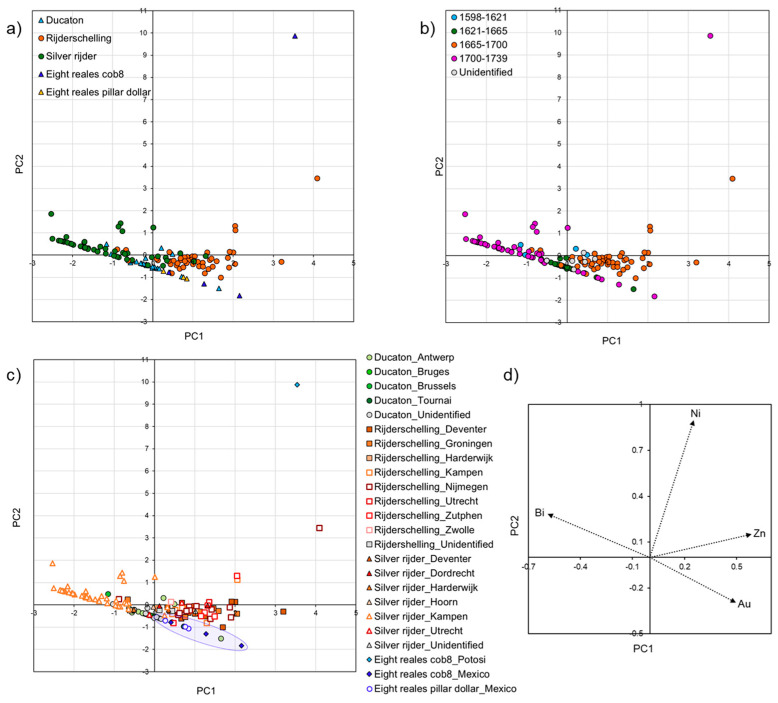
Principal component analysis (PCA) plots of the trace elemental (nickel—Ni; zinc—Zn; gold—Au; and bismuth—Bi) composition of coins from Dutch Republic, the Spanish Netherlands and a selection of coins from Spanish America (rijderschelling, silver rijder, ducatons, eight reales cob8, eight reales pillar dollars, four reales cob4 and four reales half pillar dollars) analysed in this study. The graphs represent the data based on typology (**a**), sovereign and date (**b**), location of the mint (**c**) and variables (trace elements) represented as vectors (**d**).

To gain more knowledge about the sources of silver in coins minted in the Spanish Netherlands and in the Dutch Republic, PCA was applied to this dataset (Figure 6). The first and second principle components explain 38% and 25% of the cumulated variance, respectively.

Regarding typology, the majority of ducatons are clustered together, and they show high concentrations of gold (Figure 6a). A couple of rijderschellings and silver rijders overlap with this group.

The majority of rijderschellings form a group rich in zinc, of which some silver rijders and a couple of ducatons are also a part. Some of the coins display an intermediate pattern, indicating the use of different silver stocks.

Finally, a cluster formed by silver rijders, a few rijderschellings and a couple of ducatons, which contain high bismuth, can be also observed.

In terms of chronology, the earliest coins (1598–1621) are richer in zinc and bismuth (Figure 6b). Starting from 1621, an increased number of coins shows high gold content, but some coins are still rich in zinc and bismuth, denoting once again the use of several silver suppliers in the researched European mints over time. A special cluster includes silver rijders rich in bismuth and minted in 1739.

To evaluate the silver suppliers in different mints, the dataset was divided based on location of the mints (Figure 6c). For comparison, reference coins minted in Mexico (blue symbols surrounded by a blue circle) and Potosí (light blue symbol) were added.

Ducatons were minted in the Spanish Netherlands (Antwerp, Bruges, Brussels and Tournai), and their fingerprint indicates that to produce most of the coins, silver from Mexico was used. Some coins minted in Antwerp and Brussels show a source rich in zinc and bismuth (most probably of central European origin). Most of the coins with an unidentified mint were struck with Mexican silver.

The rijderschellings were produced in the Dutch Republic in Deventer, Groningen, Harderwijk, Kampen, Nijmegen, Utrecht, Zutphen and Zwolle. The dataset did not allow a clear separation of coins minted in these mints, as the data overlap. Most of the coins contain a high zinc level, which was also found in coins minted in Germany (Nuremberg), while others have significant amounts of bismuth, which was observed in coins struck in Saxony [57]. The results also indicate that some of the coins were manufactured by using different suppliers of silver, in particular from Mexico (rich in gold) and from Potosí (due to traces of nickel). Similarly to other coins minted in Germany, these coins contain zinc, bismuth and nickel. The latter might have been introduced during recycling and alloying with copper/copper alloys from central European ores.

The silver rijders were also produced in mints in the Dutch Republic (Deventer, Dordrecht, Harderwijk, Hoorn, Kampen and Utrecht) by using different sources of silver as for the rijderschellings. An exception is represented by a large number of silver rijders, which were minted in Kampen (Overijssel) in 1739 and exhibit a high concentration of bismuth, which was found in high amounts in coins minted in Saxony [57]. This result proves that, despite the decrease in silver production in European mines in the 17th century [15,41] and the increase of the influx of American silver, silver from central Europe was still mined and traded in the Dutch mints [35,57,59].

When comparing silver suppliers, a higher number of coins produced in the mints in the Spanish Netherlands used Mexican sources, proving that the higher availability of American silver was available here compared to the Dutch Republic. In addition, only a few coins from the European mints contain the fingerprint of silver from Potosí (traces of nickel, but not tin and antimony), which is probably due to the decrease in silver extraction in this area after 1650 [16,17,29,35,51,75].

## 4. Discussion

In this study, trace elemental analysis was carried out on a selection of coins recovered from the *Rooswijk* shipwreck to provide further knowledge of silver circulation and suppliers in mints in the Northern and Southern Netherlands. While previous works focused on the study of coins struck in the Americas, Portugal and Spain, with only a few examples of coins from the Northern and Southern Netherlands, this research focuses on the analysis of coins minted in the Low Countries over a wide timeframe (1618–1739).

SEM-EDS investigations proved that the analysis of corroded silver coins with silver content higher than 91 wt% is broadly representative of the bulk, unless severe corrosion can be observed.

A total of 275 coins were further studied by µXRF to reveal any changes in their trace elemental fingerprint according to typology, date, and location of the mint.

### 4.1. Coin Circulation and Trade

The results of this study provided further knowledge on coin circulation and the types of coins used for the Asian market.

Different types of coins were on board, including larger (ducatons, silver rijders, eight reales cob8, eight reales pillar dollars, four reales cob4 and four reales half pillar dollars) and smaller (rijderschellings) coins, which were minted in Mexico, Potosí (Bolivia), the Spanish Netherlands and the Dutch Republic.

The group of ducatons include the oldest coins, and, together with the silver rijders, they show the widest range of dates. On the contrary, the rijderschellings were struck in a short period of time (1673–1692). These coins were exported in high quantities to Asia [12,13].

Finally, the dates of production of the coins minted in Mexico were quite narrow and relatively recent, as they were struck between 1724 and 1738, suggesting a short timespan between production in the Americas and reminting and use for overseas trade after arriving in Europe.

### 4.2. Impact of American Silver in European Coinage

The study of the trace elements of the coins showed the impact of American silver in European coinage. Among the analysed American coins in the *Rooswijk*, only two can be attributed to Potosí (Bolivia), while the others were minted in Mexico.

The compositional studies indicate that silver from Mexican ores is rich in gold, while silver from Potosí contains more nickel, confirming previous studies on silver minted in the Americas [24,29,51,57,58,59]. The elemental fingerprint of three coins possibly minted in Lima overlap with Mexican coins, highlighting issues in discriminating these different silver sources. Unfortunately, these three coins are too worn to make a certain attribution.

Most of the coins produced in the Dutch Republic display a trace elemental fingerprint, which differs from the silver from the American ores, as they show higher amounts of zinc and bismuth, which were found in high concentrations in coins struck in Germany [57]. These elements were introduced in silver from a silver ore or, especially for zinc, from copper sources used in the alloy after refining or recycling. While some coins were struck using pure silver from Mexico, it seems that most of the coins also contain silver from Europe. The re-melting and recycling of older coins minted with European silver or the use of new central European silver had an impact on the formation of a trace element fingerprint distinctive from the pure sources [36]. This result indicates that silver from central Europe was still traded in the Netherlands, despite the high influx of silver from the Americas and the decrease in silver production in Europe in the 17th century [15,41], and it is consistent with other compositional studies of Dutch silver coins [35,57,59]. In the assemblage, a large number of silver rijders minted in Kampen in 1739 was found. These coins form a specific subgroup in the compositional studies, due to their high levels of bismuth, which is associated with coins minted in Saxony [57]. This result further confirms that high amounts of silver were imported from central Europe to produce new coins. These coins were probably acquired directly by traders from Dutch mints without being circulated locally. For instance, it was a common practice to supply vast amounts of silver rijders for the VOC [11]. In the 18th century, no trade embargoes between Spain and the Dutch Republic were imposed, but the decreasing role of the Dutch Republic in world trade could have led to their new focus on central Europe to import silver [76,77]. The mechanism of remelting old European silver to produce new coins was always active in the background.

Compared to the Dutch Republic, most of the coins minted in the Spanish Netherlands used Mexican sources, indicating a wider circulation of American silver in this territory. In addition, almost none of the coins minted in Europe displayed the fingerprint of Potosian silver. This could be due to the decrease in silver extraction in Potosí after 1650 and, conversely, the increased production in Mexico [16,17,29,35,51,75].

Clearly, not only silver bars but also high amounts of coins minted both in the Americas and in the Netherlands were exported to Batavia by the VOC for trading purposes. In particular, the records of the Bookkeeper General of the VOC in Batavia show that in the 18th century, hundreds of ships were registered as having transported silver (zilver) from the Republic to Batavia [78]. The higher number of coins minted in Mexico than in Potosí recovered from the site shows the decrease in Potosían production and the increase in the production and trade of Mexican silver. The most interesting outcome of this study is that, despite the high inflow of silver from the Americas, the Dutch mints still relied at least partly on the recycling of older coins minted with European silver and on the import of silver from central Europe.

## 5. Conclusions

Micro-XRF spectroscopy combined with SEM-EDS analysis of the coins from the cargo of the VOC vessel *Rooswijk* proved to be suitable to study their trace elemental composition, morphology and state of preservation. Minimally destructive compositional studies based on trace elements of the coins provided further knowledge on the use of different silver suppliers in the Spanish Netherlands and in the Dutch Republic, which are not associated with a specific silver mine. This research represents one of the first studies of a large corpus of coins minted in the Low Countries over a wide timeframe (1618–1739).

The results of this study highlight that

- Most of the coins struck in the mints in the Dutch Republic exhibit a specific trace elemental fingerprint, which is the result of the re-melting and recycling of older coins minted with European silver, new silver from central European sources and silver from the Americas. This result proves the impact of the influx of Spanish-American silver in Europe and the special role of central European silver.
- Almost all the coins from the Americas were minted in Mexico, supporting other studies which proved a decrease in silver extraction in Potosí after 1650.
- The study contributed to the production of a reference tool to identify different silver suppliers to produce coins whose mint location could not be identified due to their poor state of preservation.

Further analysis of coins struck in other mints in Europe and in the Americas, combined with isotope analysis and investigations with techniques able to provide additional trace elements, will improve the accuracy in the identification of the provenance of silver suppliers, unlocking information about world trade and silver movement over time.

## Figures and Tables

**Table 1:** Summary of the data obtained by SEM-EDS (wt%) analyses of the bulk and surface of the coins. The tabulated results are averages of three analyses and normalised.

Small Finds Number	Coin Type			Cu	Ag	Pb
RK17A00280	Four reales cob4	bulk	Average	2.51	96.93	0.48
			St. Dev.	0.16	0.31	0.03
		surface	Average	1.77	97.88	0.17
			St. Dev.	0.43	0.73	0.30
RK17A00422	Four reales cob4	bulk	Average	4.09	95.55	0.36
			St. Dev.	2.35	2.21	0.38
		surface	Average	6.37	93.39	0.24
			St. Dev.	4.57	4.47	0.21
RK17A00333	Eight reales cob8	bulk	Average	2.29	97.71	0.00
			St. Dev.	2.01	2.01	0.00
		surface	Average	5.72	93.70	0.57
			St. Dev.	2.81	3.05	0.54
RK17A00361	Eight reales cob8	bulk	Average	4.77	95.08	0.15
			St. Dev.	1.44	1.67	0.27
		surface	Average	12.51	86.95	0.54
			St. Dev.	3.93	4.12	0.47
RK17A02853	Ducaton	bulk	Average	4.78	95.09	0.13
			St. Dev.	0.72	0.75	0.27
		surface	Average	3.42	95.98	0.60
			St. Dev.	0.69	0.61	0.09
RK17A02959	Ducaton	bulk	Average	4.61	94.86	0.53
			St. Dev.	0.42	0.33	0.09
		surface	Average	4.48	94.89	0.63
			St. Dev.	0.54	0.67	0.13
RK17A00689	Silver rijder	bulk	Average	4.11	95.48	0.41
			St. Dev.	0.78	0.62	0.37
		surface	Average	2.78	96.84	0.38
			St. Dev.	1.13	0.93	0.33
RK17A02785	Silver rijder	bulk	Average	4.52	94.64	0.84
			St. Dev.	0.80	0.88	0.20
		surface	Average	4.13	94.98	0.89
			St. Dev.	0.50	0.53	0.63
RK17A00096	Eight reales pillar dollar	bulk	Average	3.44	96.44	0.13
			St. Dev.	0.42	0.41	0.26
		surface	Average	2.34	97.55	0.11
			St. Dev.	2.41	2.32	0.22
RK17A00842	Rijderschelling	bulk	Average	7.74	91.72	0.53
			St. Dev.	1.58	1.89	0.31
		surface	Average	6.02	93.53	0.45
			St. Dev.	0.14	0.01	0.13
RK17A00877	Rijderschelling	bulk	Average	18.23	80.84	0.93
			St. Dev.	4.94	4.82	0.11
		surface	Average	6.92	92.00	1.07
			St. Dev.	1.40	1.47	0.09

## Data Availability

The data presented in this study are included in the article/Supplementary Material. Further inquiries can be directed to the corresponding author.

## Supplementary Materials

The following supporting information can be downloaded at https://www.mdpi.com/article/10.3390/ma18050925/s1, Figure S1: SEM images (BSE detector) of cross-sections from four reales cob4 (RK17A00422, bulk, (a)), silver rijder (RK17A02785, (b)), rijderschelling (RK17A00877, (c)) and eight reales pillar dollar (RK17A00096, bulk, (d)); Figure S2: Main, minor and trace elements (µXRF, wt%) in the coins of the assemblage; Figure S3: Ternary diagrams of copper—Cu; silver—Ag; and lead—Pb (a) and of gold—Au; zinc—Zn; and bismuth—Bi (b) (µXRF, wt%) in the coins of the assemblage. Figure S4: Principal component analysis (PCA) plots of the trace elemental (nickel—Ni; zinc—Zn; gold—Au; and bismuth—Bi) composition of the coins minted in Mexico, Potosi and Spanish America analysed in this study. The graphs represent the data based on typology (a), sovereign (b), provenance (c) and variables (trace elements) represented as vectors (d). In the graph representing the provenance, yellow symbols are coins (RK17A00624, RK17A00972 and RK17A01023) identified as possibly minted in Santa Fé (de Bogota) or Lima; Figure S5: Principal component analysis (PCA) plots of the trace elemental (nickel—Ni; zinc—Zn; gold—Au; and bismuth—Bi) composition of the coins minted in Mexico (eight reales cob8, eight reales pillar dollars, four reales cob4 and four reales half pillar dollars) analysed in this study. The graphs represent the data based on typology (a), date (b) and variables (trace elements) represented as vectors (c). Table S1: Summary of the silver coins analysed by micro X-ray fluorescence (µXRF) spectroscopy. When mint of origin was not visible, the possible mint of origin according to PCA is added; Table S2: µXRF analysis of certified reference materials of known composition (wt%). The tabulated results are averages of three analyses, normalised and compared to known values (bd = below detection); Table S3: Chemical composition (µXRF, wt%) of the coins (bd = below detection). The tabulated results are averages of at least three analyses and normalised.
