# Supplementary material for: Investigating the Sources of Silver in 17th- and 18th-Century Silver Coins from the Rooswijk Shipwreck by Compositional Studies
Source: Materials (Basel). 2025 Feb 20;18(5):925. doi: 10.3390/ma18050925 (PMC11901266; doi:10.3390/ma18050925)
Supplement: Supplementary file 1 [file materials-18-00925-s001.zip › materials-3405888-supplementary.pdf]

Supporting Information

Investigating the Sources of Silver in 17th- and 18th-Century Silver Coins from the Rooswijk Shipwreck by Compositional Studies

Francesca Gherardi [1,*] and Jan Pelsdonk [2,3]

[1] Investigative Science, Historic England, Fort Cumberland, Fort Cumberland Rd, PO4 9LD, Portsmouth, United Kingdom.

[2] Rijksmuseum, Amsterdam 1071 XX, The Netherlands

[3] Teylers Museum, Haarlem 2011 CH, The Netherlands; duit@live.nl

[*] Corresponding author: francesca.gherardi@historicengland.org.uk

1. Results

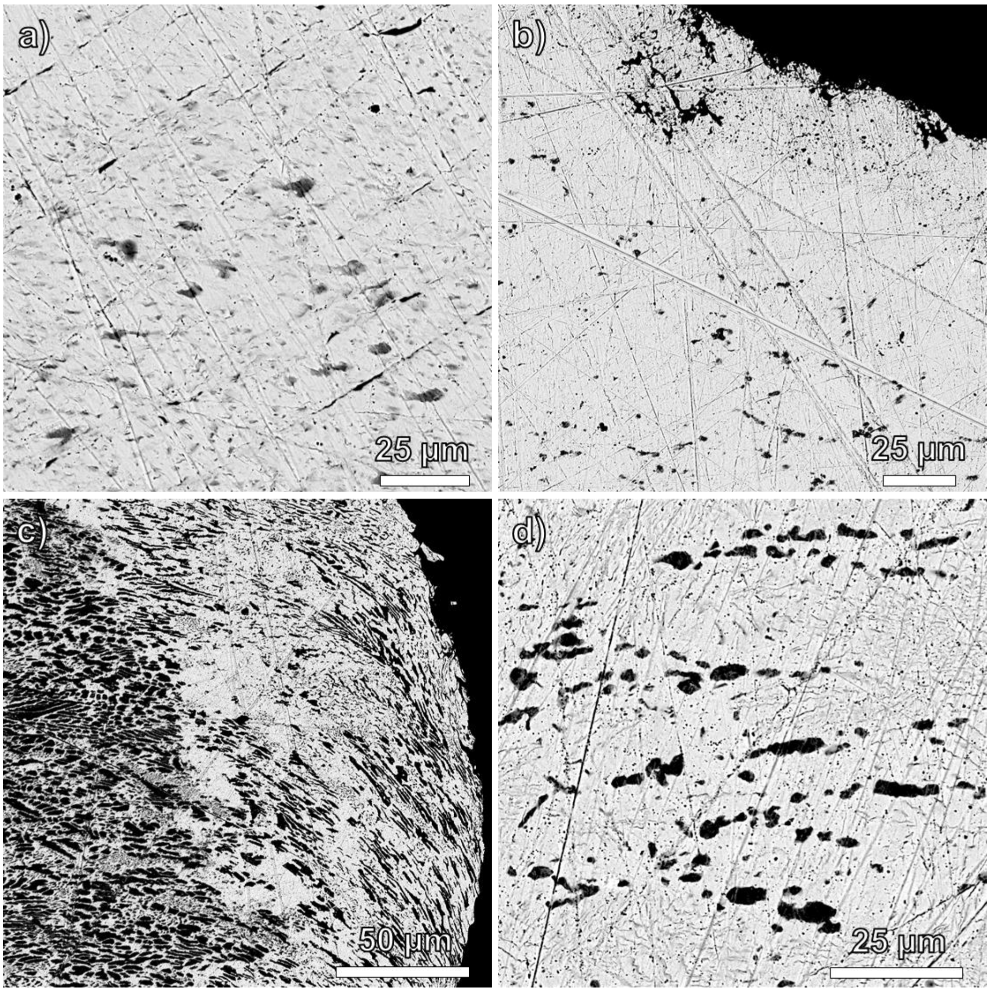

Figure S1. SEM images (BSE detector) of cross-sections from four reales cob4 (RK17A00422, bulk, a.), silver rijder (RK17A02785, b.), rijderschelling (RK17A00877, c.) and eight reales pillar dollar (RK17A00096, bulk, d.).

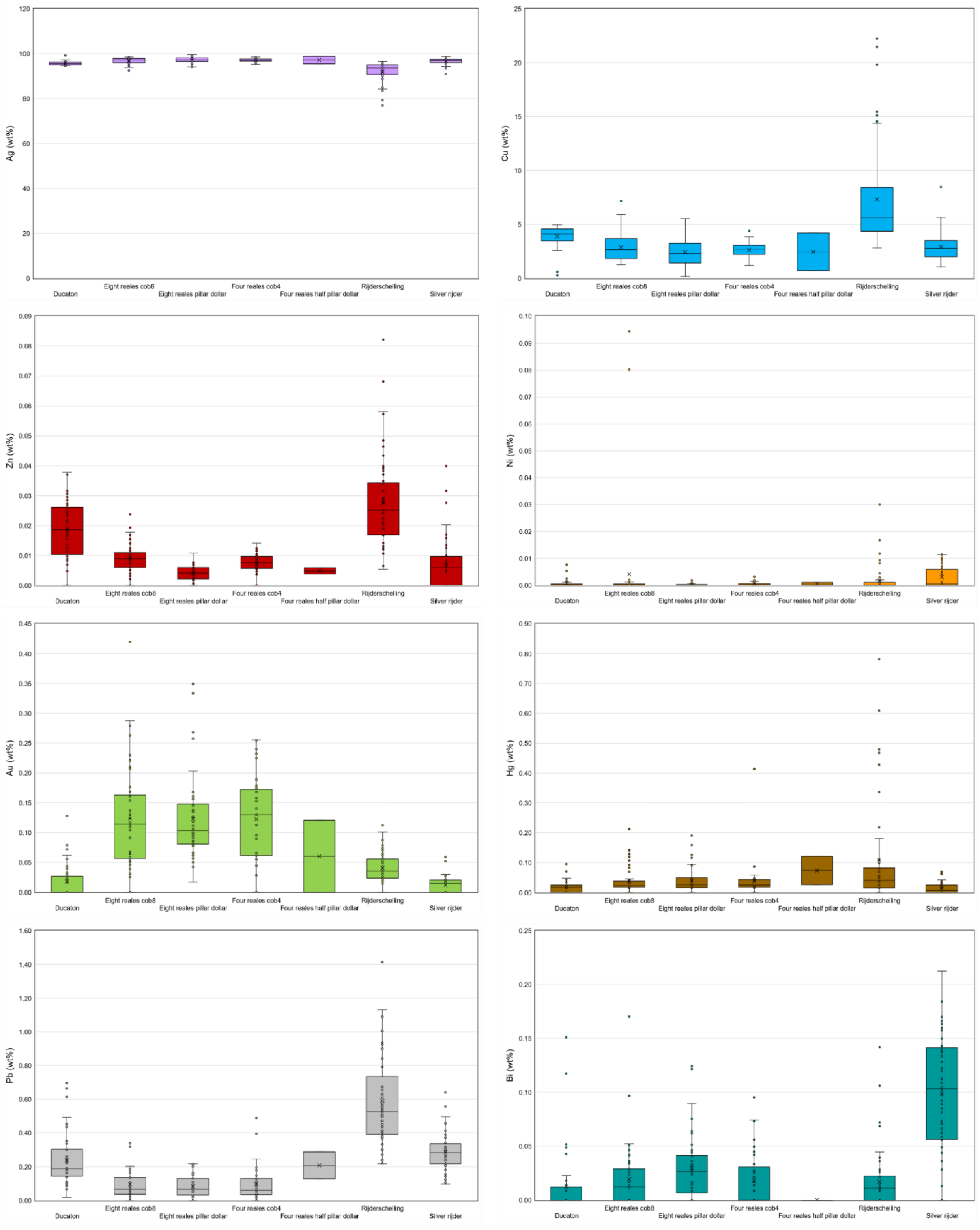

Figure S2. Main, minor and trace elements (µXRF, wt%) in the coins of the assemblage.

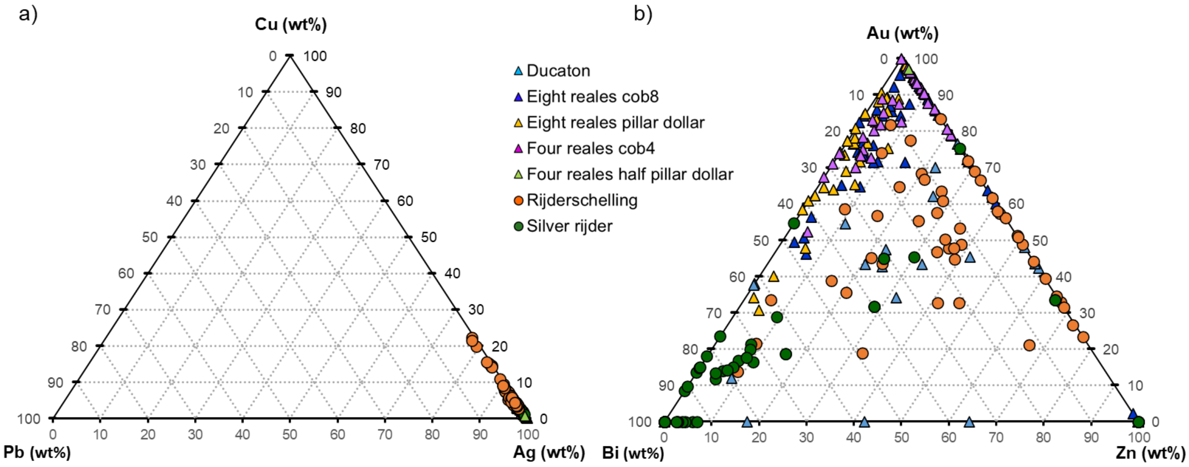

Figure S3. Ternary diagrams of copper-Cu, silver-Ag and lead-Pb (a) and of gold-Au, zinc-Zn and bismuth-Bi (b) (µXRF, wt%) in the coins of the assemblage.

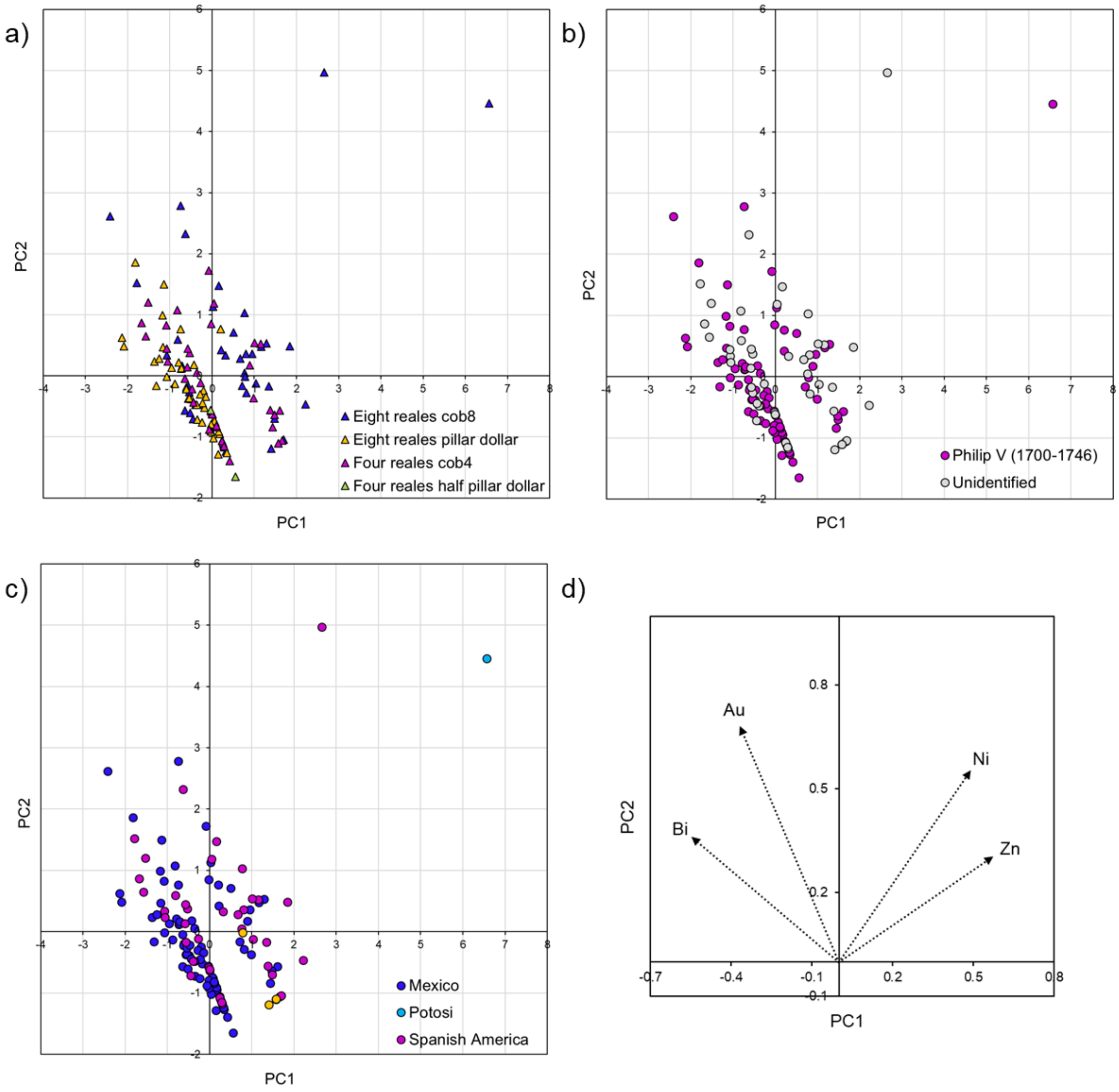

Figure S4. Principal component analysis (PCA) plots of the trace elemental (nickel-Ni, zinc-Zn, gold-Au and bismuth-Bi) composition of the coins minted in Mexico, Potosi and Spanish America analysed in this study. The graphs represent the data based on typology (a), sovereign (b), provenance (c) and variables (trace elements) represented as vectors (d). In the graph representing the provenance, yellow symbols are coins (RK17A00624, RK17A00972 and RK17A01023) identified as possibly been minted in Santa Fé (de Bogota) or Lima.

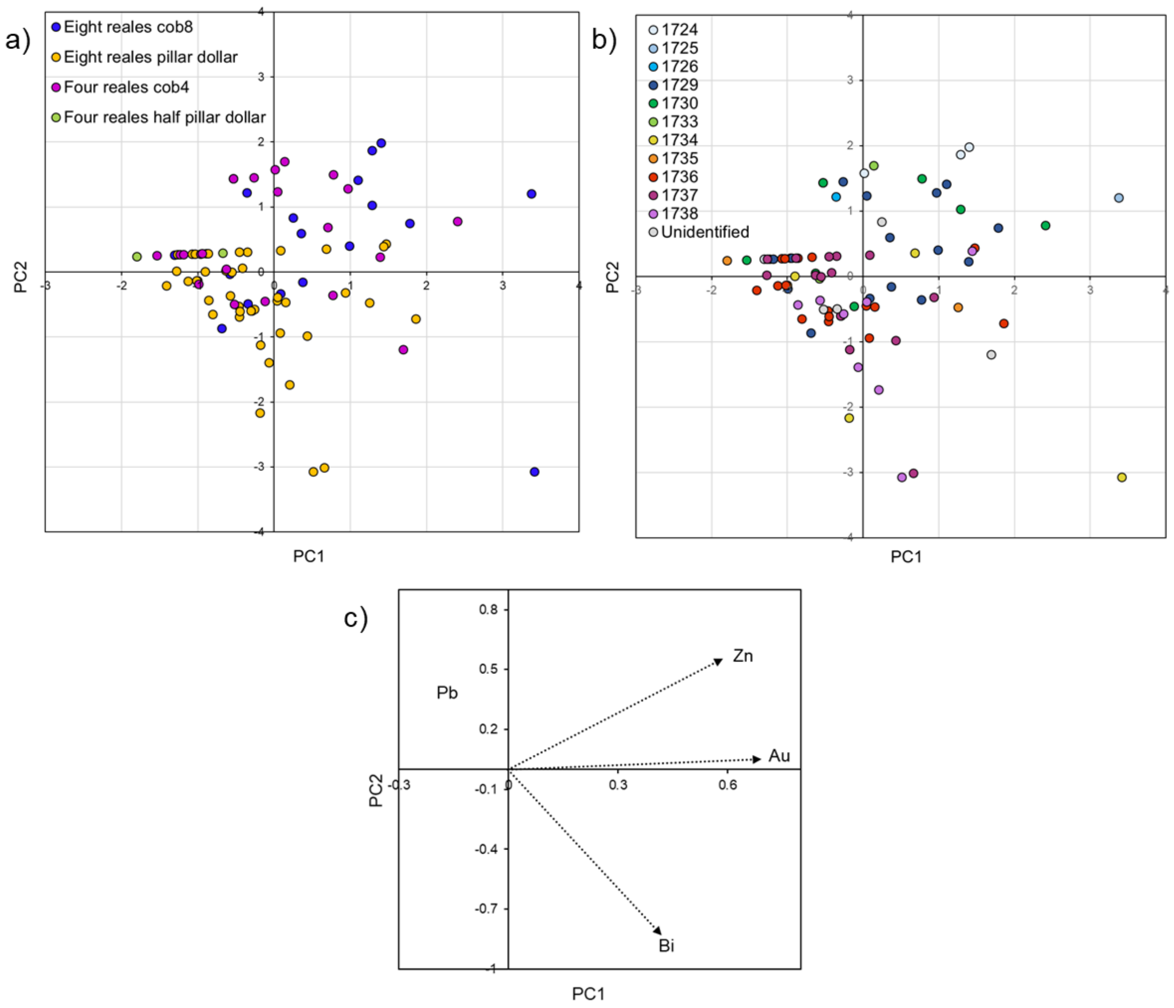

Figure S5. Principal component analysis (PCA) plots of the trace elemental (nickel-Ni, zinc-Zn, gold-Au and bismuth-Bi) composition of the coins minted in Mexico (eight reales cob8, eight reales pillar dollars, four reales cob4 and four reales half pillar dollars) analysed in this study. The graphs represent the data based on typology (a), date (b) and variables (trace elements) represented as vectors (c).

Table S1. Summary of the silver coins analysed by micro X-ray Fluorescence (μXRF) spectroscopy. When mint of origin was not visible, the possible mint of origin according to PCA is added.

Small find number	Type	Date	Country of origin	Mint of origin	Mint of origin according to PCA	Sovereign	Assayer's Mark
RK17 A00096	Eight reales pillar dollar	1737	Viceroyalty of New Spain	Mexico		Philip V	Manuel de la Pena & Francisco de la Peña Flores
RK17 A00097	Eight reales cob8	(1700-1734)	Viceroyalty of New Spain	Mexico		Philip V	Not visible
RK17 A00098	Eight reales cob8	Not visible	Spanish America	Not visible	Mexico	Not visible	Not visible
RK17 A00099	Eight reales pillar dollar	1737	Viceroyalty of New Spain	Mexico		Philip V	Manuel de la Pena & Francisco de la Peña Flores
RK17 A00104	Eight reales pillar dollar	1737	Viceroyalty of New Spain	Mexico		Philip V	Manuel de la Pena & Francisco de la Peña Flores
RK17 A00111	Four reales cob4	Not visible	Spanish America	Mexico		Not visible	Not visible
RK17 A00112	Eight reales cob8	Not visible	Spanish America	Not visible	Mexico	Not visible	Not visible
RK17 A00116	Four reales cob4	Not visible	Spanish America	Not visible	Mexico	Not visible	Not visible
RK17 A00117	Eight reales cob8	1725	Viceroyalty of New Spain	Mexico		Philip V	José de Rivas Angulo
RK17 A00120	Eight reales pillar dollar	1736	Viceroyalty of New Spain	Mexico		Philip V	Manuel de la Pena & Francisco de la Peña Flores
RK17 A00121	Four reales cob4	Not visible	Spanish America	Not visible	Mexico	Not visible	Not visible
RK17 A00145	Eight reales pillar dollar	1736	Viceroyalty of New Spain	Mexico		Philip V	Manuel de la Pena & Francisco de la Peña Flores
RK17 A00146	Eight reales pillar dollar	1737	Viceroyalty of New Spain	Mexico		Philip V	Manuel de la Pena & Francisco de la Peña Flores
RK17 A00147	Eight reales pillar dollar	1736	Viceroyalty of New Spain	Mexico		Philip V	Manuel de la Pena & Francisco de la Peña Flores
RK17 A00154	Eight reales cob8	Not visible	Spanish America	Not visible	Mexico	Not visible	Not visible
RK17 A00160	Eight reales pillar dollar	1734	Viceroyalty of New Spain	Mexico		Philip V	Manuel de la Pena & Francisco de la Peña Flores
RK17 A00171	Four reales cob4	1730	Viceroyalty of New Spain	Mexico		Philip V	Nicholas de Roxas or José de Rivas Angulo
RK17 A00172	Eight reales cob8	Not visible, 1729-1730	Viceroyalty of New Spain	Mexico		Philip V	Nicholas de Roxas or José de Rivas Angulo
RK17 A00175	Eight reales cob8	Not visible	Spanish America	Not visible	Mexico	Not visible	Not visible
RK17 A00197	Eight reales cob8	Not visible, 1729-1730	Viceroyalty of New Spain	Mexico		Philip V	Nicholas de Roxas or José de Rivas Angulo
RK17 A00200	Four reales cob4	Not visible	Spanish America	Not visible	Mexico	Not visible	Not visible

RK17 A00238	Eight reales pillar dollar	1736	Viceroyalty of New Spain	Mexico		Philip V	Manuel de la Pena & Francisco de la Peña Flores
RK17 A00239	Eight reales pillar dollar	1736	Viceroyalty of New Spain	Mexico		Philip V	Manuel de la Pena & Francisco de la Peña Flores
RK17 A00240	Eight reales pillar dollar	1738	Viceroyalty of New Spain	Mexico		Philip V	Manuel de la Pena & Francisco de la Peña Flores
RK17 A00241	Eight reales pillar dollar	1737	Viceroyalty of New Spain	Mexico		Philip V	Manuel de la Pena & Francisco de la Peña Flores
RK17 A00243	Four reales cob4	1733	Viceroyalty of New Spain	Mexico		Philip V	Felipe Rivas de Angulo
RK17 A00245	Eight reales cob8	Not visible	Viceroyalty of New Spain	Mexico		Not visible	Not visible
RK17 A00248	Eight reales pillar dollar	1736	Viceroyalty of New Spain	Mexico		Philip V	Manuel de la Pena & Francisco de la Peña Flores
RK17 A00249	Eight reales pillar dollar	1738	Viceroyalty of New Spain	Mexico		Philip V	Manuel de la Pena & Francisco de la Peña Flores
RK17 A00256	Four reales half pillar dollar	1736	Viceroyalty of New Spain	Mexico		Philip V	Manuel de la Pena & Francisco de la Peña Flores
RK17 A00265	Four reales cob4	1730	Viceroyalty of New Spain	Mexico		Philip V	Nicholas de Roxas or José de Rivas Angulo
RK17 A00266	Four reales cob4	Not visible	Spanish America	Not visible	Mexico	Not visible	Not visible
RK17 A00267	Eight reales cob8	1724-1729	Viceroyalty of New Spain	Mexico		Philip V	José de Rivas Angulo
RK17 A00271	Rijderschelling	169* (1690-1691)	United Netherlands	Nijmegen		Nijmegen	Mintmaster Gerard van Harn
RK17 A00280	Four reales cob4	1729-1730	Viceroyalty of New Spain	Mexico		Philip V	Nicholas de Roxas or José de Rivas Angulo
RK17 A00281	Four reales cob4	Not visible	Viceroyalty of New Spain	Not visible	Mexico	Not visible	Not visible
RK17 A00282	Four reales cob4	Not visible	Spanish America	Not visible	Mexico	Not visible	Not visible
RK17 A00283	Four reales cob4	1730	Viceroyalty of New Spain	Mexico		Philip V	Nicholas de Roxas or José de Rivas Angulo
RK17 A00284	Four reales cob4	1729-1730	Viceroyalty of New Spain	Mexico		Philip V	Nicholas de Roxas or José de Rivas Angulo
RK17 A00290	Four reales cob4	1724-1729	Viceroyalty of New Spain	Mexico		Philip V	José de Rivas Angulo
RK17 A00291	Four reales cob4	1729-1730	Viceroyalty of New Spain	Mexico		Philip V	Nicholas de Roxas or José de Rivas Angulo
RK17 A00295	Four reales cob4	Not visible	Spanish America	Not visible	Mexico	Not visible	Not visible
RK17 A00297	Four reales cob4	1729-1730	Viceroyalty of New Spain	Mexico		Philip V	Nicholas de Roxas or José de Rivas Angulo
RK17 A00300	Four reales cob4	1730	Viceroyalty of New Spain	Mexico		Philip V	Nicholas de Roxas or José de Rivas Angulo
RK17 A00306	Four reales cob4	1729-1730	Viceroyalty of New Spain	Mexico		Philip V	Nicholas de Roxas or José de Rivas Angulo

RK17 A00309	Four reales cob4	Not visible	Viceroyalty of New Spain	Mexico		Not visible	Not visible
RK17 A00314	Four reales cob4	1729-1730	Viceroyalty of New Spain	Mexico		Philip V	Nicholas de Roxas or José de Rivas Angulo
RK17 A00315	Four reales cob4	1730	Viceroyalty of New Spain	Mexico		Philip V	Nicholas de Roxas or José de Rivas Angulo
RK17 A00323	Eight reales cob8	1729-1730	Viceroyalty of New Spain	Mexico		Philip V	Nicholas de Roxas or José de Rivas Angulo
RK17 A00324	Eight reales cob8	Not visible	Viceroyalty of New Spain	Mexico		Not visible	Not visible
RK17 A00327	Eight reales cob8	1730	Viceroyalty of New Spain	Mexico		Philip V	Nicholas de Roxas or José de Rivas Angulo
RK17 A00331	Eight reales cob8	1729-1730	Viceroyalty of New Spain	Mexico		Philip V	Nicholas de Roxas or José de Rivas Angulo
RK17 A00333	Eight reales cob8	Not visible	Spanish America	Not visible	Mexico	Not visible	Not visible
RK17 A00343	Eight reales cob8	Not visible	Spanish America	Not visible	Mexico	Not visible	Not visible
RK17 A00344	Eight reales cob8	1729-1730	Viceroyalty of New Spain	Mexico		Philip V	Nicholas de Roxas or José de Rivas Angulo
RK17 A00347	Eight reales cob8	Not visible	Spanish America	Not visible	Mexico	Not visible	Not visible
RK17 A00355	Eight reales cob8	Not visible	Spanish America	Not visible	Mexico	Not visible	Not visible
RK17 A00360	Eight reales cob8	Not visible	Spanish America	Not visible	Mexico	Not visible	Not visible
RK17 A00361	Eight reales cob8	1729	Viceroyalty of New Spain	Mexico		Philip V	Nicholas de Roxas or José de Rivas Angulo
RK17 A00391	Eight reales cob8	Not visible	Spanish America	Not visible	Potosi	Not visible	Not visible
RK17 A00393	Eight reales pillar dollar	1738	Viceroyalty of New Spain	Mexico		Philip V	Manuel de la Pena & Francisco de la Peña Flores
RK17 A00395	Eight reales pillar dollar	1737	Viceroyalty of New Spain	Mexico		Philip V	Manuel de la Pena & Francisco de la Peña Flores
RK17 A00396	Eight reales pillar dollar	1736	Viceroyalty of New Spain	Mexico		Philip V	Manuel de la Pena & Francisco de la Peña Flores
RK17 A00400	Eight reales pillar dollar	1737	Viceroyalty of New Spain	Mexico		Philip V	Manuel de la Pena & Francisco de la Peña Flores
RK17 A00401	Eight reales pillar dollar	1737	Viceroyalty of New Spain	Mexico		Philip V	Manuel de la Pena & Francisco de la Peña Flores
RK17 A00402	Eight reales pillar dollar	1737	Viceroyalty of New Spain	Mexico		Philip V	Manuel de la Pena & Francisco de la Peña Flores
RK17 A00407	Eight reales pillar dollar	1737	Viceroyalty of New Spain	Mexico		Philip V	Manuel de la Pena & Francisco de la Peña Flores
RK17 A00422	Four reales cob4	1729-1730	Viceroyalty of New Spain	Mexico		Philip V	Nicholas de Roxas or José de Rivas Angulo
RK17 A00443	Four reales cob4	Not visible	Spanish America	Not visible	Mexico	Not visible	Not visible

RK17 A00448	Four reales cob4	1729-1730	Viceroyalty of New Spain	Mexico		Philip V	Nicholas de Roxas or José de Rivas Angulo
RK17 A00458	Silver rijder	1674	United Netherlands	Utrecht		Utrecht	Mintmaster Johan van Reynevelt or Adriaan van der Heyden
RK17 A00462	Half ducaton	1618-1639	Spanish Netherlands	Not visible		Albert & Isabella or Philip IV	Not visible
RK17 A00478	Eight reales cob8	Not visible	Spanish America	Not visible	Mexico	Not visible	Not visible
RK17 A00509	Eight reales cob8	1724-1729	Viceroyalty of New Spain	Mexico		Philip V	José de Rivas Angulo
RK17 A00514	Eight reales cob8	Not visible	Spanish America	Not visible	Mexico	Not visible	Not visible
RK17 A00547	Eight reales pillar dollar	1736	Viceroyalty of New Spain	Mexico		Philip V	Manuel de la Pena & Francisco de la Peña Flores
RK17 A00563	Eight reales pillar dollar	1737	Viceroyalty of New Spain	Mexico		Philip V	Manuel de la Pena & Francisco de la Peña Flores
RK17 A00570	Eight reales pillar dollar	1735	Viceroyalty of New Spain	Mexico		Philip V	Manuel de la Pena & Francisco de la Peña Flores
RK17 A00576	Eight reales cob8	1726?	Viceroyalty of New Spain	Mexico		Philip V	Not visible
RK17 A00582	Eight reales cob8	1724-1730	Viceroyalty of New Spain	Mexico		Philip V	José de Rivas Angulo or Nicholas de Roxas
RK17 A00585	Eight reales cob8	1705	Viceroyalty of Peru	Potosí		Philip V	Diego de Ybarbouru
RK17 A00589	Four reales cob4	1730?	Viceroyalty of New Spain	Mexico		Philip V	Not visible
RK17 A00593	Four reales cob4	1730	Viceroyalty of New Spain	Mexico		Philip V	Nicholas de Roxas or José de Rivas Angulo
RK17 A00600	Eight reales cob8	Not visible	Spanish America	Not visible	Mexico	Not visible	Not visible
RK17 A00623	Four reales cob4	1724-1729	Viceroyalty of New Spain	Mexico		Philip V	José de Rivas Angulo
RK17 A00624	Eight reales cob8	1723	Viceroyalty of Peru	Lima	Mexico	Philip V	Not visible
RK17 A00629	Rijderschelling	1688	United Netherlands	Zutphen		Zutphen	Mintmaster Herman van Bayen
RK17 A00633	Rijderschelling	1691	United Netherlands	Groningen		Groningen	Mintmaster Gerard van Harn
RK17 A00634	Rijderschelling	1680-1691	United Netherlands	Kampen?		Kampen?	Not visible
RK17 A00651	Rijderschelling	1690-1692	United Netherlands	Groningen		Groningen	Mintmaster Egbert Marinus
RK17 A00664	Rijderschelling	1686	United Netherlands	Nijmegen		Nijmegen	Mintmaster Gerard van Harn
RK17 A00666	Rijderschelling	1691	United Netherlands	Deventer		Deventer	Mintmaster Pieter Sluysken
RK17 A00677	Rijderschelling	Not visible	United Netherlands	Not visible		Not visible	Not visible
RK17 A00689	Silver rijder	Not visible, 1659-1677	United Netherlands	Kampen		Overijssel	Not visible
RK17 A00691	Silver rijder	Not visible, 1659-1738	United Netherlands	Enkhuizen, Hoorn, or Medemblik		West-Friesland	Not visible

RK17 A00692	Silver rijder	Not visible, 1659-1668	United Netherlands	Dordrecht		Holland	Mintmaster Simon Rottermond
RK17 A00696	Silver rijder	1692	United Netherlands	Utrecht		Utrecht	Mintmaster Johan van Romondt
RK17 A00700	Four reales cob4	Not visible	Spanish America	Not visible	Mexico	Not visible	Not visible
RK17 A00707	Four reales cob4	Not visible	Spanish America	Not visible	Mexico	Not visible	Not visible
RK17 A00713	Eight reales pillar dollar	1736	Viceroyalty of New Spain	Mexico		Philip V	Manuel de la Pena & Francisco de la Peña Flores
RK17 A00734	Eight reales cob8	1729	Viceroyalty of New Spain	Mexico		Philip V	Nicholas de Roxas or José de Rivas Angulo
RK17 A00749	Ducaton	Not visible, 1618-1639	Spanish Netherlands	Antwerp or Brussels		Philip IV	Not visible
RK17 A00765	Four reales cob4	Not visible	Spanish America	Not visible	Mexico	Not visible	Not visible
RK17 A00783	Rijderschelling	1691	United Netherlands	Deventer		Deventer	Mintmaster Pieter Sluysken
RK17 A00796	Rijderschelling	1691	United Netherlands	Groningen		Groningen	Mintmaster Egbert Marinus
RK17 A00797	Rijderschelling	1680-1691	United Netherlands	Kampen		Kampen	Mintmaster Jacob Ridder
RK17 A00799	Rijderschelling	1686	United Netherlands	Deventer		Deventer	Mintmaster Pieter Sluysken
RK17 A00811	Rijderschelling	1686	United Netherlands	Nijmegen		Nijmegen	Mintmaster Gerard van Harn
RK17 A00851	Rijderschelling	1688	United Netherlands	Zutphen		Zutphen	Mintmaster Herman van Bayen
RK17 A00853	Rijderschelling	1691	United Netherlands	Groningen		Groningen and Ommelanden	Mintmaster Assuerus Vosdink
RK17 A00854	Rijderschelling	1686	United Netherlands	Deventer		Deventer	Mintmaster Pieter Sluysken
RK17 A00857	Rijderschelling	1679-1691	United Netherlands	Kampen		Overijssel	Mintmaster Dirk van Romondt senior
RK17 A00870	Rijderschelling	Not visible, 1673-1692	United Netherlands	Not visible		Not visible	Not visible
RK17 A00877	Rijderschelling	1688	United Netherlands	Zutphen		Zutphen	Mintmaster Herman van Bayen
RK17 A00888	Rijderschelling	1690-1691	United Netherlands	Kampen		Kampen	Mintmaster Jacob Ridder
RK17 A00889	Rijderschelling	1691	United Netherlands	Kampen		Overijssel	Mintmaster Dirk van Romondt senior
RK17 A00896	Rijderschelling	1690	United Netherlands	Kampen		Overijssel	Mintmaster Dirk van Romondt senior
RK17 A00902	Rijderschelling	1690-1692	United Netherlands	Groningen		Groningen and Ommelanden	Mintmaster Assuerus Vosdink
RK17 A00903	Rijderschelling	1691	United Netherlands	Deventer		Deventer	Mintmaster Pieter Sluysken
RK17 A00910	Rijderschelling	1689	United Netherlands	Nijmegen		Nijmegen	Mintmaster Gerard van Harn
RK17 A00911	Rijderschelling	1686	United Netherlands	Nijmegen		Nijmegen	Mintmaster Gerard van Harn
RK17 A00912	Rijderschelling	1683?	United Netherlands	Deventer		Deventer	Mintmaster Pieter Sluysken
RK17 A00915	Rijderschelling	1689	United Netherlands	Nijmegen		Nijmegen	Mintmaster Gerard van Harn

RK17 A00951	Eight reales cob8	Not visible	Spanish America	Not visible	Mexico	Not visible	Not visible
RK17 A00952	Eight reales cob8	Not visible	Spanish America	Not visible	Mexico	Not visible	Not visible
RK17 A00953	Eight reales cob8	Not visible	Spanish America	Not visible	Mexico	Not visible	Not visible
RK17 A00957	Eight reales cob8	Not visible	Spanish America	Not visible	Mexico	Not visible	Not visible
RK17 A00958	Eight reales cob8	Not visible	Spanish America	Not visible	Mexico	Not visible	Not visible
RK17 A00959	Four reales cob4	Not visible	Spanish America	Not visible	Mexico	Not visible	Not visible
RK17 A00960	Four reales cob4	Not visible	Spanish America	Not visible	Mexico	Not visible	Not visible
RK17 A00963	Four reales cob4	Not visible	Spanish America	Not visible	Mexico	Not visible	Not visible
RK17 A00972	Eight reales cob8	171(0?)	Viceroyalty of Peru	Lima	Mexico	Not visible	Not visible
RK17 A00993	Rijderschelling	Not visible, 1681-1691	United Netherlands	Harderwijk		Guelders	Mintmaster Pieter Sluysken or Johan van Brienen
RK17 A01023	Eight reales cob8	Not visible	Viceroyalty of Peru	Lima	Mexico	Not visible	Not visible
RK17 A01049	Silver rijder	1676	United Netherlands	Kampen		Kampen	Mintmaster Jacob Ridder
RK17 A01050	Ducaton	1657	Spanish Netherlands	Antwerp		Philip IV	Mintmaster Caspar Antheunis or George de Bruyn van Aelst
RK17 A01062	Eight reales pillar dollar	1736	Viceroyalty of New Spain	Mexico		Philip V	Manuel de la Pena & Francisco de la Peña Flores
RK17 A01077	Rijderschelling	1685	United Netherlands	Nijmegen		Nijmegen	Mintmaster Gerard van Harn
RK17 A01079	Eight reales pillar dollar	1737	Viceroyalty of New Spain	Mexico		Philip V	Manuel de la Pena & Francisco de la Peña Flores
RK17 A01082	Eight reales cob8	Not visible	Spanish America	Not visible	Mexico	Not visible	Not visible
RK17 A01089	Four reales cob4	1730	Viceroyalty of New Spain	Mexico		Philip V	Nicholas de Roxas or José de Rivas Angulo
RK17 A01092	Eight reales pillar dollar	1737	Viceroyalty of New Spain	Mexico		Philip V	Manuel de la Pena & Francisco de la Peña Flores
RK17 A01093	Eight reales pillar dollar	1736	Viceroyalty of New Spain	Mexico		Philip V	Manuel de la Pena & Francisco de la Peña Flores
RK17 A01099	Eight reales pillar dollar	1734	Viceroyalty of New Spain	Mexico		Philip V	Manuel de la Pena & Francisco de la Peña Flores
RK17 A01101	Four reales cob4	Not visible	Spanish America	Not visible	Mexico	Not visible	Not visible
RK17 A01107	Eight reales cob8	Not visible	Spanish America	Not visible	Mexico	Not visible	Not visible
RK17 A01109	Eight reales cob8	Not visible	Spanish America	Not visible	Mexico	Not visible	Not visible
RK17 A01111	Eight reales cob8	Not visible	Spanish America	Not visible	Mexico	Not visible	Not visible
RK17 A01112	Eight reales cob8	Not visible	Spanish America	Not visible	Mexico	Not visible	Not visible
RK17 A01114	Eight reales cob8	Not visible	Spanish America	Not visible	Mexico	Not visible	Not visible

RK17 A01156	Four reales half pillar dollar	1735	Viceroyalty of New Spain	Mexico		Philip V	Manuel de la Pena & Francisco de la Peña Flores
RK17 A01182	Eight reales pillar dollar	1738	Viceroyalty of New Spain	Mexico		Philip V	Manuel de la Pena & Francisco de la Peña Flores
RK17 A01184	Eight reales pillar dollar	1738	Viceroyalty of New Spain	Mexico		Philip V	Manuel de la Pena & Francisco de la Peña Flores
RK17 A01187	Eight reales pillar dollar	1738	Viceroyalty of New Spain	Mexico		Philip V	Manuel de la Pena & Francisco de la Peña Flores
RK17 A01189	Eight reales pillar dollar	1737	Viceroyalty of New Spain	Mexico		Philip V	Manuel de la Pena & Francisco de la Peña Flores
RK17 A01190	Eight reales pillar dollar	1737	Viceroyalty of New Spain	Mexico		Philip V	Manuel de la Pena & Francisco de la Peña Flores
RK17 A01204	Eight reales pillar dollar	1736	Viceroyalty of New Spain	Mexico		Philip V	Manuel de la Pena & Francisco de la Peña Flores
RK17 A01208	Eight reales pillar dollar	1736	Viceroyalty of New Spain	Mexico		Philip V	Manuel de la Pena & Francisco de la Peña Flores
RK17 A01214	Eight reales pillar dollar	1736	Viceroyalty of New Spain	Mexico		Philip V	Manuel de la Pena & Francisco de la Peña Flores
RK17 A01219	Eight reales pillar dollar	1736	Viceroyalty of New Spain	Mexico		Philip V	Manuel de la Pena & Francisco de la Peña Flores
RK17 A01239	Ducaton	1619	Spanish Netherlands	Antwerp		Albert and Isabella	Not visible
RK17 A01241	Eight reales cob8	1733	Viceroyalty of New Spain	Mexico		Philip V	Felipe Rivas de Angulo
RK17 A01249	Four reales cob4	1730	Viceroyalty of New Spain	Mexico		Philip V	Nicholas de Roxas or José de Rivas Angulo
RK17 A01270	Ducaton	1679	Spanish Netherlands	Antwerp		Charles II	Mintmaster George de Bruyn van Aelst
RK17 A01286	Ducaton	1636	Spanish Netherlands	Bruges		Philip IV	Mintmaster Jan van der Plancke
RK17 A01302	Ducaton	Not visible, 1618-1639	Spanish Netherlands	Antwerp or Brussels		Philip IV	Not visible
RK17 A01304	Ducaton	Half visible, 1651-1661	Spanish Netherlands	Antwerp or Brussels		Philip IV	Not visible
RK17 A01305	Ducaton	1633	Spanish Netherlands	Brussels		Philip IV	Mintmaster Gilbert Clenarts
RK17 A01313	Eight reales pillar dollar	1734	Viceroyalty of New Spain	Mexico		Philip V	Manuel de la Pena & Francisco de la Peña Flores
RK17 A01331	Ducaton	1673	Spanish Netherlands	Bruges		Charles II	Mintmaster Christoffel de Ceuninck
RK17 A02618	Silver rijder	1739	United Netherlands	Kampen		Overijssel	Mintmaster Coenraad Hendrik Cramer
RK17 A02619	Silver rijder	1739	United Netherlands	Kampen		Overijssel	Mintmaster Coenraad Hendrik Cramer
RK17 A02625	Ducaton	1655	Spanish Netherlands	Antwerp		Philip IV	Mintmaster Caspar Antheunis

RK17 A02628	Silver rijder	1739	United Netherlands	Kampen		Overijssel	Mintmaster Coenraad Hendrik Cramer
RK17 A02631	Silver rijder	1739	United Netherlands	Kampen		Overijssel	Mintmaster Coenraad Hendrik Cramer
RK17 A02632	Silver rijder	1739	United Netherlands	Kampen		Overijssel	Mintmaster Coenraad Hendrik Cramer
RK17 A02635	Silver rijder	1739	United Netherlands	Kampen		Overijssel	Mintmaster Coenraad Hendrik Cramer
RK17 A02642	Silver rijder	1739	United Netherlands	Kampen		Overijssel	Mintmaster Coenraad Hendrik Cramer
RK17 A02645	Silver rijder	1739	United Netherlands	Kampen		Overijssel	Mintmaster Coenraad Hendrik Cramer
RK17 A02651	Silver rijder	1739	United Netherlands	Kampen		Overijssel	Mintmaster Coenraad Hendrik Cramer
RK17 A02652	Silver rijder	1739	United Netherlands	Kampen		Overijssel	Mintmaster Coenraad Hendrik Cramer
RK17 A02653	Silver rijder	1739	United Netherlands	Kampen		Overijssel	Mintmaster Coenraad Hendrik Cramer
RK17 A02654	Silver rijder	1739	United Netherlands	Kampen		Overijssel	Mintmaster Coenraad Hendrik Cramer
RK17 A02655	Silver rijder	1739	United Netherlands	Kampen		Overijssel	Mintmaster Coenraad Hendrik Cramer
RK17 A02656A	Silver rijder	1739	United Netherlands	Kampen		Overijssel	Mintmaster Coenraad Hendrik Cramer
RK17 A02656B	Silver rijder	1739	United Netherlands	Kampen		Overijssel	Mintmaster Coenraad Hendrik Cramer
RK17 A02657	Silver rijder	1739	United Netherlands	Kampen		Overijssel	Mintmaster Coenraad Hendrik Cramer
RK17 A02658B	Silver rijder	1739	United Netherlands	Kampen		Overijssel	Mintmaster Coenraad Hendrik Cramer
RK17 A02659	Silver rijder	1739	United Netherlands	Kampen		Overijssel	Mintmaster Coenraad Hendrik Cramer
RK17 A02660	Silver rijder	1739	United Netherlands	Kampen		Overijssel	Mintmaster Coenraad Hendrik Cramer
RK17 A02662	Silver rijder	1739	United Netherlands	Kampen		Overijssel	Mintmaster Coenraad Hendrik Cramer
RK17 A02669	Silver rijder	1739	United Netherlands	Kampen		Overijssel	Mintmaster Coenraad Hendrik Cramer
RK17 A02671	Silver rijder	1739	United Netherlands	Kampen		Overijssel	Mintmaster Coenraad Hendrik Cramer
RK17 A02694	Silver rijder	1739	United Netherlands	Kampen		Overijssel	Mintmaster Coenraad Hendrik Cramer

RK17 A02700	Silver rijder	1739	United Netherlands	Kampen		Overijssel	Mintmaster Coenraad Hendrik Cramer
RK17 A02701	Silver rijder	1739	United Netherlands	Kampen		Overijssel	Mintmaster Coenraad Hendrik Cramer
RK17 A02713	Silver rijder	1739	United Netherlands	Kampen		Overijssel	Mintmaster Coenraad Hendrik Cramer
RK17 A02724	Silver rijder	1739	United Netherlands	Kampen		Overijssel	Mintmaster Coenraad Hendrik Cramer
RK17 A02726	Silver rijder	1739	United Netherlands	Kampen		Overijssel	Mintmaster Coenraad Hendrik Cramer
RK17 A02727	Silver rijder	1739	United Netherlands	Kampen		Overijssel	Mintmaster Coenraad Hendrik Cramer
RK17 A02729	Silver rijder	1739	United Netherlands	Kampen		Overijssel	Mintmaster Coenraad Hendrik Cramer
RK17 A02733	Ducaton	1673	Spanish Netherlands	Antwerp or Brussels		Charles II	Not visible
RK17 A02740	Silver rijder	1739	United Netherlands	Kampen		Overijssel	Mintmaster Coenraad Hendrik Cramer
RK17 A02741	Silver rijder	1739	United Netherlands	Kampen		Overijssel	Mintmaster Coenraad Hendrik Cramer
RK17 A02750	Silver rijder	1739	United Netherlands	Kampen		Overijssel	Mintmaster Coenraad Hendrik Cramer
RK17 A02755	Ducaton	1656	Spanish Netherlands	Antwerp or Brussels		Philip IV	Not visible
RK17 A02760	Silver rijder	1739	United Netherlands	Kampen		Overijssel	Mintmaster Coenraad Hendrik Cramer
RK17 A02771	Ducaton	Half visible, 1647-1657	Spanish Netherlands	Antwerp or Brussels		Philip IV	Not visible
RK17 A02775	Silver rijder	1739?	United Netherlands	Kampen		Overijssel	Mintmaster Coenraad Hendrik Cramer
RK17 A02780	Ducaton	Half visible, 1650-1659	Spanish Netherlands	Antwerp or Brussels		Philip IV	Not visible
RK17 A02785	Silver rijder	1739	United Netherlands	Kampen		Overijssel	Mintmaster Coenraad Hendrik Cramer
RK17 A02788	Silver rijder	1666	United Netherlands	Kampen		Overijssel	Mintmaster Johan van Harn
RK17 A02798	Silver rijder	1739	United Netherlands	Kampen		Overijssel	Mintmaster Coenraad Hendrik Cramer
RK17 A02801	Silver rijder	1739	United Netherlands	Kampen		Overijssel	Mintmaster Coenraad Hendrik Cramer
RK17 A02819	Ducaton	1664	Spanish Netherlands	Antwerp		Philip IV	Mintmaster George de Bruyn van Aelst
RK17 A02820	Silver rijder	1670	United Netherlands	Harderwijk		Guelders	Mintmaster Paulus Sluysken
RK17 A02821B	Ducaton	1648	Spanish Netherlands	Antwerp		Philip IV	Mintmaster Caspar Antheunis

RK17 A02822	Silver rijder	1668	United Netherlands	Dordrecht		Holland	Mintmaster Simon Rottermond
RK17 A02823	Ducaton	1673	Spanish Netherlands	Brussels		Charles II	Mintmaster George de Bruyn van Aelst
RK17 A02824E	Ducaton	1664	Spanish Netherlands	Antwerp or Burssels		Philip IV	Not visible
RK17 A02825	Ducaton	Not visible, 1618-1639	Spanish Netherlands	Not visible		Albert & Isabella or Philip IV	Not visible
RK17 A02826	Ducaton	Half visible, 1619-1639	Spanish Netherlands	Not visible		Albert & Isabella or Philip IV	Not visible
RK17 A02827	Ducaton	Not visible, 1618-1639	Spanish Netherlands	Not visible		Albert & Isabella or Philip IV	Not visible
RK17 A02829A	Ducaton	1670	Spanish Netherlands	Bruges		Philip IV	Mintmaster Christoffel de Ceuninck
RK17 A02831	Ducaton	1641	Spanish Netherlands	Antwerp		Philip IV	Mintmaster Leonard Damery or Gilbert Clenaerts
RK17 A02840	Silver rijder	1664	United Netherlands	Deventer		Deventer	Mintmaster Johan van Harn or Willem Sluysken
RK17 A02842	Ducaton	1636	Spanish Netherlands	Brussels		Philip IV	Mintmaster Gilbert Clenarts
RK17 A02843	Ducaton	1665	Spanish Netherlands	Brussels		Philip IV	Mintmaster Pieter van Vreeckem
RK17 A02844	Ducaton	Half visible, 1660-1669	Spanish Netherlands	Bruges		Philip IV	Mintmaster Christoffel de Ceuninck
RK17 A02845	Ducaton	1654	Spanish Netherlands	Antwerp		Philip IV	Mintmaster Caspar Antheunis
RK17 A02846	Ducaton	1650	Spanish Netherlands	Antwerp		Philip IV	Mintmaster Caspar Antheunis
RK17 A02847	Silver rijder	1662	United Netherlands	Hoorn		West-Friesland	Mintmaster Gerrit van Romondt
RK17 A02849	Ducaton	Not visible, 1618-1639	Spanish Netherlands	Not visible		Philip IV	Not visible
RK17 A02850	Ducaton	1634	Spanish Netherlands	Not visible		Philip IV	Not visible
RK17 A02851	Ducaton	Half visible, 1642-1648	Spanish Netherlands	Antwerp		Philip IV	Mintmaster Gilbert Clenaerts or Caspar Antheunis
RK17 A02852	Ducaton	Not visible, 1618-1639	Spanish Netherlands	Antwerp or Brussels		Philip IV	Not visible
RK17 A02853	Ducaton	1618	Spanish Netherlands	Brussels		Albert & Isabella	Mintmaster Pieter van der Heyden
RK17 A02859A	Ducaton	1619	Spanish Netherlands	Antwerp		Albert & Isabella	Mintmaster Dominique Wouters or Jeanne van Liebeke
RK17 A02860C	Ducaton	1650	Spanish Netherlands	Antwerp		Philip IV	Mintmaster Caspar Antheunis
RK17 A02860D	Ducaton	1704	Spanish Netherlands	Antwerp		Philip V	Mintmaster Jean Baptiste Sneyers senior
RK17 A02861	Ducaton	1652	Spanish Netherlands	Antwerp		Philip IV	Mintmaster Caspar Antheunis
RK17 A02862	Ducaton	1650	Spanish Netherlands	Antwerp		Philip IV	Mintmaster Caspar Antheunis

RK17 A02864	Ducaton	1647	Spanish Netherlands	Tournai		Philip IV	Mintmaster Antoine de la Derrière
RK17 A02865	Ducaton	1636	Spanish Netherlands	Brussels		Philip IV	Mintmaster Gilbert Clenarts
RK17 A02872	Ducaton	Half visible, 1618-1699	Spanish Netherlands	Not visible		Not visible	Not visible
RK17 A02874	Rijderschelling	1689?	United Netherlands	Deventer		Deventer	Mintmaster Pieter Sluysken
RK17 A02875	Rijderschelling	1690	United Netherlands	Deventer		Deventer	Mintmaster Pieter Sluysken
RK17 A02878	Rijderschelling	1688	United Netherlands	Zutphen		Zutphen	Mintmaster Herman van Bayen
RK17 A02893	Rijderschelling	1691	United Netherlands	Nijmegen		Nijmegen	Mintmaster Gerard van Harn
RK17 A02896	Rijderschelling	1688	United Netherlands	Nijmegen		Nijmegen	Mintmaster Gerard van Harn
RK17 A02897	Rijderschelling	1689	United Netherlands	Deventer		Deventer	Mintmaster Pieter Sluysken
RK17 A02898	Rijderschelling	1686	United Netherlands	Deventer		Deventer	Mintmaster Pieter Sluysken
RK17 A02901	Rijderschelling	1691	United Netherlands	Nijmegen		Nijmegen	Mintmaster Gerard van Harn
RK17 A02908	Rijderschelling	1690	United Netherlands	Kampen		Kampen	Mintmaster Jacob Ridder
RK17 A02909	Rijderschelling	1689	United Netherlands	Zwolle		Zwolle	Mintmaster Cornelis van Keppel Fox
RK17 A02910	Rijderschelling	1686	United Netherlands	Nijmegen		Nijmegen	Mintmaster Gerard van Harn
RK17 A02911	Rijderschelling	1686	United Netherlands	Utrecht		Utrecht	Mintmaster Johan van Romondt
RK17 A02915	Rijderschelling	1691?	United Netherlands	Harderwijk		Guelders	Mintmaster Johan van Brienen
RK17 A02917	Rijderschelling	1691	United Netherlands	Groningen		Groningen	Mintmaster Egbert Marinus
RK17 A02922	Rijderschelling	1688	United Netherlands	Zwolle		Zwolle	Mintmaster Cornelis van Keppel Fox
RK17 A02933	Rijderschelling	1686	United Netherlands	Deventer		Deventer	Mintmaster Pieter Sluysken
RK17 A02935	Rijderschelling	Half visible, 1683-1683	United Netherlands	Deventer		Deventer	Mintmaster Pieter Sluysken
RK17 A02936	Rijderschelling	1688?	United Netherlands	Kampen		Overijssel	Mintmaster Dirk van Romondt senior
RK17 A02937	Rijderschelling	1688	United Netherlands	Zutphen		Zutphen	Mintmaster Herman van Bayen
RK17 A02938	Rijderschelling	1686	United Netherlands	Deventer		Deventer	Mintmaster Pieter Sluysken
RK17 A02942	Rijderschelling	169-	United Netherlands	Groningen		Groningen	Mintmaster Egbert Marinus
RK17 A02947	Rijderschelling	1689	United Netherlands	Zutphen		Zutphen	Mintmaster Herman van Bayen
RK17 A02959	Ducaton	1636	Spanish Netherlands	Antwerp or Brussels		Philip IV	Not visible
RK18 A00128	Eight reales cob8	1733	Viceroyalty of New Spain	Mexico		Philip V	Felipe Rivas de Angulo
RK18 A00129	Eight reales pillar dollar	1738	Viceroyalty of New Spain	Mexico		Philip V	Manuel de la Pena & Francisco de la Peña Flores
RK18 A00230	Rijderschelling	1688	United Netherlands	Nijmegen		Nijmegen	Mintmaster Gerard van Harn
RK18 A00239	Ducaton	1636	Spanish Netherlands	Brussels		Philip IV	Mintmaster Gilbert Clenarts

RK18 A00241	Eight reales pillar dollar	1738	Viceroyalty of New Spain	Mexico		Philip V	Manuel de la Pena & Francisco de la Peña Flores
RK18 A00259	Rijderschelling	1691	United Netherlands	Groningen		Groningen and Ommelanden	Mintmaster Assuerus Vosdink
RK18 A00268	Ducaton	1652	Spanish Netherlands	Antwerp or Brussels		Philip IV	Not visible

Table S2. μXRF analysis of certified reference materials of known composition (wt%). The tabulated results are averages of three analyses, normalised and compared to known values (bd = below detection).

Sample			Ni	Cu	Zn	Ag	Au	Pd	Sn	Pb	Fe	As	Sb	Bi	Mn	P	Si	Al
Gold standard 0707-03	Measured	Average	5.08	15.16	2.08	2.77	74.91	bd	bd	bd	bd	bd	bd	bd	bd	bd	bd	bd
		St. Dev.	0.04	0.03	0.01	0.05	0.06	bd	bd	bd	bd	bd	bd	bd	bd	bd	bd	bd
	Known		5.04	15.13	2.06	2.75	75.02	bd	bd	bd	bd	bd	bd	bd	bd	bd	bd	bd
Silver standard 1303-03	Measured	Average	bd	4.94	2.35	92.71	bd	bd	bd	bd	bd	bd	bd	bd	bd	bd	bd	bd
		St. Dev.	bd	0.04	0.05	0.11	bd	bd	bd	bd	bd	bd	bd	bd	bd	bd	bd	bd
	Known		bd	4.92	2.32	92.76	bd	bd	bd	bd	bd	bd	bd	bd	bd	bd	bd	bd
Silver standard 0732-16	Measured	Average	bd	9.52	bd	56.97	33.51	bd	bd	bd	bd	bd	bd	bd	bd	bd	bd	bd
		St. Dev.	bd	0.07	bd	0.12	0.06	bd	bd	bd	bd	bd	bd	bd	bd	bd	bd	bd
	Known		bd	9.44	bd	57.1	33.46	bd	bd	bd	bd	bd	bd	bd	bd	bd	bd	bd
Gold standard 0744-16	Measured	Average	bd	9.48	1.55	27.05	55.73	6.19	bd	bd	bd	bd	bd	bd	bd	bd	bd	bd
		St. Dev.	bd	0.02	0.02	0.08	0.07	0.04	bd	bd	bd	bd	bd	bd	bd	bd	bd	bd
	Known		bd	9.43	1.52	26.80	55.68	6.57	bd	bd	bd	bd	bd	bd	bd	bd	bd	bd
Copper alloy standard C71.31	Measured	Average	1.99	83.51	4.14	0.05	bd	bd	3.79	6.20	0.10	0.08	0.06	0.05	0.01	0.01	0.01	bd
		St. Dev.	0.02	0.04	0.02	0.01	bd	bd	0.02	0.03	0.01	0.01	0.01	0.01	0.01	0.01	0.01	bd
	Known		2.05	83.34	4.01	0.04	bd	bd	4.01	6.25	0.06	0.06	0.08	0.06	0.02	0.01	0.00	bd
Copper alloy standard C71.34	Measured	Average	0.01	87.87	1.12	0.07	bd	bd	7.77	2.53	0.27	0.16	0.11	0.01	0.05	0.01	0.01	0.01
		St. Dev.	0.01	0.05	0.04	0.01	bd	bd	0.05	0.02	0.02	0.01	0.01	0.01	0.01	0.01	0.01	0.01
	Known		0.01	87.80	1.10	0.06	bd	bd	7.81	2.59	0.22	0.13	0.15	0.02	0.07	0.01	0.02	0.01
Copper alloy standard B10	Measured	Average	0.99	83.82	2.81	bd	bd	bd	6.82	3.95	0.21	0.01	1.11	bd	bd	0.01	bd	0.28
		St. Dev.	0.02	0.05	0.05	bd	bd	bd	0.08	0.03	0.02	0.01	0.01	bd	bd	0.01	bd	0.01
	Known		1.01	83.59	2.77	0.00	bd	bd	7.00	4.07	0.17	0.01	1.14	bd	bd	0.01	bd	0.22
Copper alloy standard B22	Measured	Average	0.18	83.48	14.79	bd	bd	bd	0.21	0.12	0.11	0.15	0.17	0.18	0.12	0.10	0.12	0.26
		St. Dev.	0.02	0.06	0.07	bd	bd	bd	0.01	0.01	0.02	0.01	0.01	0.02	0.01	0.02	0.01	0.01
	Known		0.18	83.58	14.63	bd	bd	bd	0.19	0.15	0.10	0.14	0.17	0.22	0.15	0.14	0.15	0.21
Copper alloy standard GM4	Measured	Average	2.04	82.91	7.39	0.01	bd	bd	2.54	4.92	0.07	0.04	0.05	0.03	bd	bd	bd	bd
		St. Dev.	0.02	0.04	0.07	0.01	bd	bd	0.03	0.02	0.01	0.01	0.01	0.01	bd	bd	bd	bd
	Known		2.06	82.85	7.19	0.01	bd	bd	2.51	5.22	0.05	0.02	0.04	0.04	0.00	0.01	0.01	bd

Table S3. Chemical composition (µXRF, wt%) of the coins (bd = below detection). The tabulated results are averages of at least three analyses and normalised.

Small find number		Fe	Ni	Cu	Zn	Ag	Au	Hg	Pb	Bi
RK17 A00096	Average	bd	bd	4.18	0.01	95.56	0.06	0.09	0.10	bd
	St.Dev.	bd	bd	0.82	0.00	0.82	0.01	0.03	0.03	bd
RK17 A00097	Average	0.92	bd	3.19	0.01	95.32	0.17	0.13	0.09	0.17
	St.Dev.	0.36	bd	0.44	0.00	0.27	0.03	0.01	0.03	0.02
RK17 A00098	Average	0.56	bd	1.82	0.01	97.45	0.09	0.02	0.04	bd
	St.Dev.	0.34	bd	0.22	0.01	0.58	0.01	0.00	0.02	bd
RK17 A00099	Average	bd	bd	2.55	bd	96.99	0.16	0.09	0.21	bd
	St.Dev.	bd	bd	0.29	bd	0.34	0.01	0.01	0.06	bd
RK17 A00104	Average	0.19	bd	0.19	bd	99.42	0.10	0.09	bd	bd
	St.Dev.	0.04	bd	0.18	bd	0.23	0.02	0.03	bd	bd
RK17 A00111	Average	0.28	bd	2.94	0.01	96.42	0.18	0.06	0.10	0.02
	St.Dev.	0.31	bd	0.33	0.00	0.29	0.02	0.02	0.01	0.00
RK17 A00112	Average	0.94	bd	1.30	0.02	97.63	0.03	0.04	0.05	bd
	St.Dev.	0.31	bd	0.28	0.00	0.45	0.00	0.01	0.01	bd
RK17 A00116	Average	0.25	bd	2.84	0.01	96.46	0.18	bd	0.19	0.07
	St.Dev.	0.28	bd	0.49	0.00	0.40	0.02	bd	0.05	0.01
RK17 A00117	Average	1.60	bd	1.58	0.01	96.27	0.41	0.04	0.08	0.01
	St.Dev.	1.23	bd	0.48	0.00	1.71	0.10	0.02	0.11	0.01
RK17 A00120	Average	0.12	bd	2.72	bd	96.73	0.33	0.02	0.04	0.04
	St.Dev.	0.16	bd	0.36	bd	0.42	0.04	0.01	0.01	0.01
RK17 A00121	Average	0.21	bd	2.93	0.01	96.55	0.22	0.02	0.06	bd
	St.Dev.	0.17	bd	0.58	0.00	0.46	0.01	0.00	0.02	bd
RK17 A00145	Average	0.42	bd	5.50	0.01	93.80	0.14	0.00	0.09	0.04
	St.Dev.	0.30	bd	1.64	0.00	2.00	0.01	0.00	0.04	0.01
RK17 A00146	Average	0.05	bd	2.30	bd	97.25	0.11	0.04	0.22	0.03
	St.Dev.	0.03	bd	0.27	bd	0.27	0.01	0.01	0.01	0.00
RK17 A00147	Average	0.03	bd	2.65	0.01	97.09	0.10	0.05	0.07	bd
	St.Dev.	0.03	bd	1.67	0.00	1.62	0.02	0.04	0.02	bd
RK17 A00154	Average	0.31	bd	1.83	0.01	97.67	0.09	0.02	0.04	0.03
	St.Dev.	0.10	bd	0.27	0.00	0.17	0.00	0.00	0.00	0.01
RK17 A00160	Average	bd	bd	5.11	0.01	94.65	0.04	0.03	0.07	0.09
	St.Dev.	bd	bd	0.24	0.00	0.25	0.01	0.02	0.00	0.01
RK17 A00171	Average	1.01	bd	3.48	0.01	95.19	0.14	0.02	0.15	0.01
	St.Dev.	1.02	bd	1.27	0.01	1.07	0.01	0.01	0.08	0.00
RK17 A00172	Average	1.15	bd	2.09	0.01	96.48	0.13	0.02	0.10	0.01
	St.Dev.	0.51	bd	0.42	0.00	0.78	0.02	0.00	0.04	0.01
RK17 A00175	Average	1.12	bd	2.22	0.02	96.13	0.21	0.21	bd	0.10
	St.Dev.	0.42	bd	0.26	0.00	0.73	0.02	0.02	bd	0.04
RK17 A00197	Average	0.05	bd	3.64	bd	95.81	0.17	0.12	0.18	0.02
	St.Dev.	0.02	bd	0.86	bd	0.89	0.04	0.01	0.04	0.01
RK17 A00200	Average	0.04	bd	2.94	0.01	96.83	0.06	0.02	0.10	bd
	St.Dev.	0.02	bd	0.51	0.00	0.51	0.01	0.01	0.02	bd
RK17 A00238	Average	0.45	bd	1.69	0.01	97.58	0.10	0.12	0.03	0.03
	St.Dev.	0.06	bd	0.27	0.00	0.38	0.01	0.02	0.01	0.01
RK17 A00239	Average	0.15	bd	4.22	0.01	95.42	0.08	0.03	0.09	bd
	St.Dev.	0.16	bd	0.29	0.00	0.44	0.00	0.00	0.01	bd
RK17 A00240	Average	0.06	bd	5.49	0.01	94.15	0.10	bd	0.14	0.06
	St.Dev.	0.04	bd	1.13	0.00	1.13	0.01	bd	0.01	0.01
RK17 A00241	Average	bd	bd	3.72	0.01	96.09	0.04	0.09	0.04	0.01
	St.Dev.	bd	bd	0.65	0.00	0.66	0.01	0.03	0.01	0.00

Small find number		Fe	Ni	Cu	Zn	Ag	Au	Hg	Pb	Bi
RK17 A00243	Average	1.63	bd	3.58	0.01	94.58	0.04	0.03	0.13	bd
	St.Dev.	0.54	bd	0.72	0.00	0.79	0.01	0.01	0.02	bd
RK17 A00245	Average	0.12	bd	2.73	0.01	96.85	0.11	0.02	0.13	0.03
	St.Dev.	0.05	bd	1.07	0.00	1.03	0.02	0.01	0.03	0.03
RK17 A00248	Average	bd	bd	0.22	bd	99.49	0.13	0.05	0.06	0.05
	St.Dev.	bd	bd	0.07	bd	0.10	0.03	0.01	0.01	0.01
RK17 A00249	Average	0.28	bd	1.28	0.01	97.90	0.35	0.16	0.03	bd
	St.Dev.	0.07	bd	0.10	0.00	0.01	0.02	0.04	0.01	bd
RK17 A00256	Average	0.05	bd	0.72	bd	98.85	0.12	0.12	0.13	0.01
	St.Dev.	0.03	bd	0.21	bd	0.29	0.04	0.03	0.04	0.01
RK17 A00265	Average	0.03	bd	2.68	0.01	97.12	0.09	0.04	0.03	bd
	St.Dev.	0.02	bd	0.55	0.00	0.55	0.01	0.01	0.01	bd
RK17 A00266	Average	0.33	bd	1.78	0.01	97.72	0.06	0.09	bd	bd
	St.Dev.	0.03	bd	0.37	0.00	0.38	0.00	0.01	bd	bd
RK17 A00267	Average	3.31	bd	2.86	0.01	93.59	0.15	0.02	0.06	bd
	St.Dev.	1.28	bd	0.48	0.00	1.53	0.02	0.00	0.01	bd
RK17 A00271	Average	0.23	bd	3.60	0.01	95.23	0.05	0.02	0.84	0.02
	St.Dev.	0.48	bd	0.73	0.01	1.18	0.01	0.00	0.07	0.00
RK17 A00280	Average	0.04	bd	3.06	0.01	96.74	0.09	0.02	0.04	0.01
	St.Dev.	0.06	bd	0.66	0.00	0.71	0.02	0.01	0.01	0.00
RK17 A00281	Average	1.21	bd	2.15	0.01	96.38	0.09	0.02	0.10	0.03
	St.Dev.	1.36	bd	1.33	0.00	2.28	0.00	0.01	0.07	0.01
RK17 A00282	Average	0.12	bd	3.21	0.01	96.35	0.15	bd	0.08	0.07
	St.Dev.	0.06	bd	1.23	0.00	1.22	0.01	bd	0.04	0.02
RK17 A00283	Average	0.16	bd	3.81	0.01	95.79	0.19	0.02	0.02	bd
	St.Dev.	0.09	bd	2.05	0.00	2.09	0.01	0.00	0.00	bd
RK17 A00284	Average	0.30	bd	2.47	0.01	96.58	0.13	0.41	0.07	0.02
	St.Dev.	0.44	bd	1.46	0.00	1.14	0.03	0.39	0.03	0.02
RK17 A00290	Average	0.22	bd	2.26	0.01	97.38	0.06	0.05	0.02	bd
	St.Dev.	0.20	bd	0.92	0.00	0.77	0.00	0.02	0.01	bd
RK17 A00291	Average	0.20	bd	2.05	0.01	97.63	0.07	0.03	0.02	bd
	St.Dev.	0.23	bd	0.29	0.00	0.41	0.01	0.00	0.01	bd
RK17 A00295	Average	0.14	bd	2.40	0.01	97.18	0.13	0.02	0.09	0.01
	St.Dev.	0.14	bd	0.21	0.01	0.18	0.04	0.01	0.03	0.01
RK17 A00297	Average	bd	bd	3.35	0.01	96.49	0.06	0.03	0.05	0.02
	St.Dev.	bd	bd	0.99	0.00	0.99	0.01	0.02	0.01	0.01
RK17 A00300	Average	1.99	bd	2.66	0.01	94.96	0.25	0.04	0.04	0.03
	St.Dev.	0.30	bd	1.30	0.00	1.34	0.03	0.01	0.01	0.00
RK17 A00306	Average	0.97	bd	2.32	0.01	96.36	0.16	0.02	0.13	0.04
	St.Dev.	0.68	bd	1.23	0.00	1.42	0.06	0.00	0.01	0.01
RK17 A00309	Average	0.86	bd	1.18	0.01	97.53	0.11	0.03	0.18	0.09
	St.Dev.	0.37	bd	0.67	0.00	0.60	0.01	0.01	0.05	0.04
RK17 A00314	Average	0.04	bd	1.36	bd	98.43	0.09	0.05	0.03	bd
	St.Dev.	0.03	bd	0.58	bd	0.62	0.00	0.01	0.01	bd
RK17 A00315	Average	0.75	bd	4.38	0.01	94.68	bd	0.03	0.15	bd
	St.Dev.	0.23	bd	0.42	0.00	0.32	bd	0.01	0.02	bd
RK17 A00323	Average	bd	bd	1.38	bd	98.45	0.06	0.05	0.03	0.04
	St.Dev.	bd	bd	0.71	bd	0.70	0.01	0.01	0.01	0.01
RK17 A00324	Average	0.02	bd	4.16	0.01	95.57	0.07	0.02	0.14	0.02
	St.Dev.	0.02	bd	1.03	0.00	1.04	0.00	0.01	0.02	0.01
RK17 A00327	Average	0.13	bd	4.56	0.01	95.03	0.17	0.02	0.06	0.02
	St.Dev.	0.08	bd	0.53	0.00	0.62	0.02	0.01	0.04	0.02
RK17 A00331	Average	0.04	bd	4.49	0.01	95.30	0.07	0.03	0.04	0.03

Small find number		Fe	Ni	Cu	Zn	Ag	Au	Hg	Pb	Bi
	St.Dev.	0.00	bd	0.37	0.00	0.35	0.01	0.01	0.01	0.01
RK17 A00333	Average	0.38	bd	4.67	0.01	94.45	0.28	bd	0.17	0.05
	St.Dev.	0.41	bd	0.78	0.00	0.93	0.02	bd	0.02	0.02
RK17 A00343	Average	0.35	bd	3.46	0.01	95.77	0.05	0.02	0.34	0.01
	St.Dev.	0.24	bd	1.07	0.00	0.92	0.00	0.01	0.17	0.00
RK17 A00344	Average	0.42	bd	3.42	0.01	95.76	0.21	0.04	0.12	0.03
	St.Dev.	0.13	bd	0.81	0.00	0.93	0.02	0.02	0.03	0.01
RK17 A00347	Average	1.22	bd	1.89	0.01	96.62	0.11	0.02	0.09	0.03
	St.Dev.	0.31	bd	0.27	0.00	0.47	0.01	0.00	0.04	0.01
RK17 A00355	Average	2.00	bd	2.00	0.01	95.89	0.04	0.02	0.04	bd
	St.Dev.	0.74	bd	0.32	0.00	0.66	0.01	0.00	0.01	bd
RK17 A00360	Average	0.28	bd	2.87	0.01	96.63	0.11	0.02	0.08	0.01
	St.Dev.	0.14	bd	0.68	0.00	0.67	0.00	0.00	0.01	0.00
RK17 A00361	Average	2.09	bd	3.20	0.01	94.34	0.12	0.02	0.19	0.04
	St.Dev.	0.44	bd	0.42	0.00	0.46	0.01	0.00	0.01	0.00
RK17 A00391	Average	bd	0.08	1.51	0.02	97.70	0.26	0.09	0.32	0.01
	St.Dev.	bd	0.00	0.10	0.00	0.16	0.00	0.01	0.09	0.00
RK17 A00393	Average	bd	bd	2.65	bd	97.04	0.11	bd	0.13	0.08
	St.Dev.	bd	bd	0.51	bd	0.54	0.01	bd	0.02	0.01
RK17 A00395	Average	0.50	bd	1.84	0.01	97.34	0.20	0.02	0.09	0.01
	St.Dev.	0.22	bd	0.25	0.00	0.08	0.01	0.00	0.03	0.00
RK17 A00396	Average	0.03	bd	4.31	0.01	95.48	0.05	bd	0.11	0.01
	St.Dev.	0.05	bd	1.61	0.00	1.68	0.02	bd	0.05	0.01
RK17 A00400	Average	0.32	bd	1.95	0.01	97.52	0.14	0.02	0.04	0.01
	St.Dev.	0.09	bd	1.02	0.01	1.15	0.02	0.00	0.00	0.01
RK17 A00401	Average	0.03	bd	0.62	bd	98.98	0.26	0.03	0.06	0.03
	St.Dev.	0.03	bd	0.58	bd	0.50	0.08	0.01	0.03	0.03
RK17 A00402	Average	0.03	bd	1.61	bd	98.04	0.10	0.03	0.14	0.05
	St.Dev.	0.03	bd	0.39	bd	0.37	0.03	0.01	0.02	0.01
RK17 A00407	Average	0.09	bd	0.15	bd	99.53	0.14	0.05	0.02	bd
	St.Dev.	0.12	bd	0.05	bd	0.15	0.02	0.01	0.01	bd
RK17 A00422	Average	0.33	bd	1.88	bd	97.47	0.24	0.02	0.04	0.03
	St.Dev.	0.42	bd	0.41	bd	0.59	0.04	0.00	0.02	0.03
RK17 A00443	Average	0.32	bd	2.31	0.01	97.21	0.05	0.05	0.06	bd
	St.Dev.	0.24	bd	0.91	0.00	0.79	0.01	0.01	0.01	bd
RK17 A00448	Average	0.12	bd	2.36	0.01	97.42	bd	0.04	0.03	0.01
	St.Dev.	0.05	bd	1.06	0.00	1.10	bd	0.03	0.02	0.01
RK17 A00458	Average	0.03	bd	4.25	0.01	95.41	bd	0.04	0.26	bd
	St.Dev.	0.02	bd	1.11	0.00	1.08	bd	0.03	0.02	bd
RK17 A00462	Average	0.02	bd	2.58	0.01	96.97	bd	0.07	0.30	0.05
	St.Dev.	0.01	bd	0.97	0.00	0.96	bd	0.04	0.07	0.01
RK17 A00478	Average	0.32	bd	1.23	bd	98.27	0.05	0.03	0.06	0.04
	St.Dev.	0.14	bd	0.61	bd	0.55	0.02	0.01	0.03	0.02
RK17 A00509	Average	0.22	bd	2.68	0.01	96.94	0.05	0.02	0.07	bd
	St.Dev.	0.20	bd	0.10	0.00	0.27	0.00	0.00	0.01	bd
RK17 A00514	Average	0.04	bd	1.40	bd	98.23	0.12	bd	0.20	bd
	St.Dev.	0.05	bd	0.70	bd	0.67	0.01	bd	0.06	bd
RK17 A00547	Average	0.04	bd	0.29	bd	99.40	0.02	0.19	0.04	0.02
	St.Dev.	0.03	bd	0.29	bd	0.27	0.00	0.10	0.05	0.00
RK17 A00563	Average	bd	bd	1.51	bd	98.31	0.11	0.03	0.03	0.01
	St.Dev.	bd	bd	0.46	bd	0.44	0.01	0.00	0.01	0.01
RK17 A00570	Average	0.74	bd	2.07	0.01	96.94	0.13	0.02	0.03	0.06
	St.Dev.	0.67	bd	0.17	0.00	0.88	0.02	0.00	0.03	0.03

Small find number		Fe	Ni	Cu	Zn	Ag	Au	Hg	Pb	Bi
RK17 A00576	Average	0.22	bd	2.63	0.01	97.04	0.05	0.02	0.03	0.01
	St.Dev.	0.14	bd	0.70	0.00	0.56	0.00	0.00	0.01	0.00
RK17 A00582	Average	0.40	bd	4.97	0.01	94.28	0.15	0.02	0.17	bd
	St.Dev.	0.11	bd	0.78	0.00	0.80	0.01	0.00	0.04	bd
RK17 A00585	Average	0.03	0.09	2.83	0.02	96.74	0.03	0.14	0.11	0.01
	St.Dev.	0.04	0.01	1.06	0.01	0.94	0.01	0.08	0.02	0.01
RK17 A00589	Average	bd	bd	2.06	bd	97.84	0.03	0.04	0.03	bd
	St.Dev.	bd	bd	1.29	bd	1.29	0.00	0.02	0.02	bd
RK17 A00593	Average	0.84	bd	2.74	0.01	96.12	0.14	0.02	0.11	0.03
	St.Dev.	0.43	bd	0.96	0.00	1.29	0.02	0.00	0.05	0.01
RK17 A00600	Average	0.58	bd	1.86	0.01	97.49	bd	0.03	0.03	bd
	St.Dev.	0.26	bd	0.36	0.00	0.62	bd	0.01	0.01	bd
RK17 A00623	Average	0.66	bd	2.75	0.01	96.47	0.04	0.03	0.04	bd
	St.Dev.	0.23	bd	0.58	0.00	0.44	0.00	0.00	0.00	bd
RK17 A00624	Average	0.30	bd	2.33	0.01	97.16	0.13	0.02	0.05	bd
	St.Dev.	0.07	bd	0.76	0.00	0.75	0.01	0.00	0.00	bd
RK17 A00629	Average	2.26	0.01	4.24	0.05	92.98	0.02	bd	0.44	0.01
	St.Dev.	1.89	0.01	0.58	0.02	2.16	0.00	bd	0.04	0.00
RK17 A00633	Average	5.32	bd	18.77	0.04	75.00	0.06	0.05	0.75	0.01
	St.Dev.	3.87	bd	1.94	0.01	4.35	0.01	0.05	0.03	0.00
RK17 A00634	Average	0.15	0.02	4.40	0.02	95.10	0.08	bd	0.22	0.01
	St.Dev.	0.14	0.01	1.29	0.01	1.45	0.01	bd	0.05	0.00
RK17 A00651	Average	0.08	bd	14.38	0.04	84.91	0.05	bd	0.52	0.01
	St.Dev.	0.04	bd	1.86	0.01	1.86	0.01	bd	0.07	0.00
RK17 A00664	Average	0.27	bd	14.51	0.03	84.88	bd	0.04	0.27	bd
	St.Dev.	0.41	bd	1.13	0.01	1.49	bd	0.04	0.04	bd
RK17 A00666	Average	0.11	bd	3.35	0.01	95.84	0.07	0.02	0.56	0.04
	St.Dev.	0.06	bd	0.28	0.00	0.57	0.01	0.01	0.23	0.02
RK17 A00677	Average	0.03	bd	2.80	0.01	96.56	0.02	0.04	0.52	0.02
	St.Dev.	0.00	bd	0.15	0.00	0.20	0.00	0.01	0.06	0.00
RK17 A00689	Average	bd	bd	1.85	0.01	97.98	0.01	0.05	0.10	bd
	St.Dev.	bd	bd	0.34	0.00	0.28	0.00	0.02	0.05	bd
RK17 A00691	Average	0.26	bd	3.23	0.01	96.23	bd	0.07	0.19	0.01
	St.Dev.	0.04	bd	0.33	0.00	0.39	bd	0.03	0.05	0.00
RK17 A00692	Average	bd	bd	3.68	0.01	95.43	0.10	bd	0.70	0.08
	St.Dev.	bd	bd	0.68	0.00	0.70	0.02	bd	0.05	0.00
RK17 A00696	Average	0.27	bd	2.46	0.01	97.02	0.02	0.06	0.15	bd
	St.Dev.	0.21	bd	1.69	0.00	1.57	0.01	0.05	0.07	bd
RK17 A00700	Average	0.05	bd	3.70	0.01	95.81	0.14	bd	0.25	0.05
	St.Dev.	0.02	bd	0.83	0.00	0.90	0.01	bd	0.08	0.01
RK17 A00707	Average	0.14	bd	1.49	bd	97.71	0.17	bd	0.49	bd
	St.Dev.	0.11	bd	0.89	bd	0.63	0.02	bd	0.23	bd
RK17 A00713	Average	0.19	bd	0.51	bd	99.09	0.06	0.13	bd	0.03
	St.Dev.	0.11	bd	0.51	bd	0.40	0.03	0.01	bd	0.02
RK17 A00734	Average	0.13	bd	5.00	0.01	94.40	0.05	0.02	0.34	0.05
	St.Dev.	0.02	bd	0.61	0.00	0.51	0.00	0.00	0.10	0.01
RK17 A00749	Average	bd	bd	3.36	0.03	96.43	0.02	0.05	0.11	bd
	St.Dev.	bd	bd	1.01	0.01	1.01	0.00	0.03	0.02	bd
RK17 A00765	Average	0.15	bd	2.45	bd	97.05	0.23	bd	0.10	0.01
	St.Dev.	0.16	bd	0.84	bd	1.03	0.02	bd	0.03	0.00
RK17 A00783	Average	bd	bd	7.65	0.02	91.84	0.10	bd	0.38	0.01
	St.Dev.	bd	bd	1.46	0.00	1.44	0.01	bd	0.08	0.00
RK17 A00796	Average	0.04	bd	9.40	0.03	90.13	bd	0.09	0.30	bd

Small find number		Fe	Ni	Cu	Zn	Ag	Au	Hg	Pb	Bi
	St.Dev.	0.02	bd	1.08	0.00	1.05	bd	0.05	0.07	bd
RK17 A00797	Average	0.21	bd	15.07	0.03	84.09	0.03	bd	0.56	0.01
	St.Dev.	0.28	bd	1.18	0.00	1.60	0.01	bd	0.18	0.01
RK17 A00799	Average	bd	bd	9.13	0.02	90.18	0.05	0.05	0.56	0.01
	St.Dev.	bd	bd	2.97	0.00	3.00	0.00	0.03	0.06	0.00
RK17 A00811	Average	0.62	0.03	7.42	0.08	91.37	0.04	bd	0.43	0.01
	St.Dev.	0.14	0.01	1.30	0.01	1.28	0.00	bd	0.14	0.01
RK17 A00851	Average	0.02	bd	7.27	0.03	92.38	0.04	0.04	0.22	bd
	St.Dev.	0.02	bd	2.79	0.01	2.79	0.00	0.02	0.07	bd
RK17 A00853	Average	0.02	bd	7.09	0.03	91.43	0.04	0.47	0.92	bd
	St.Dev.	0.01	bd	1.34	0.00	1.00	0.01	0.09	0.26	bd
RK17 A00854	Average	0.03	bd	6.42	0.06	92.96	0.02	0.04	0.47	bd
	St.Dev.	0.02	bd	0.21	0.02	0.18	0.01	0.00	0.01	bd
RK17 A00857	Average	0.03	bd	4.83	0.01	94.14	0.02	bd	0.94	0.03
	St.Dev.	0.00	bd	1.64	0.00	1.87	0.00	bd	0.22	0.01
RK17 A00870	Average	0.03	bd	14.11	0.02	85.41	0.04	bd	0.37	0.03
	St.Dev.	0.01	bd	3.59	0.00	3.52	0.01	bd	0.07	0.01
RK17 A00877	Average	0.06	bd	5.84	0.02	93.59	0.05	0.02	0.41	0.01
	St.Dev.	0.06	bd	1.72	0.00	1.49	0.00	0.01	0.20	0.01
RK17 A00888	Average	0.48	bd	4.79	0.01	94.28	0.03	0.03	0.37	0.01
	St.Dev.	0.27	bd	2.28	0.00	2.59	0.00	0.02	0.13	0.00
RK17 A00889	Average	0.03	bd	7.71	0.02	91.04	0.03	0.22	0.94	0.01
	St.Dev.	0.02	bd	2.89	0.00	2.97	0.00	0.07	0.07	0.00
RK17 A00896	Average	bd	bd	8.36	0.01	90.69	0.07	0.18	0.67	0.01
	St.Dev.	bd	bd	1.49	0.00	1.50	0.01	0.07	0.07	0.00
RK17 A00902	Average	0.39	bd	15.40	0.03	83.16	0.03	0.08	0.90	0.02
	St.Dev.	0.63	bd	3.43	0.01	3.77	0.01	0.07	0.15	0.00
RK17 A00903	Average	bd	bd	5.61	0.01	93.93	0.02	0.08	0.34	bd
	St.Dev.	bd	bd	0.76	0.00	0.75	0.00	0.07	0.14	bd
RK17 A00910	Average	0.40	0.01	5.24	0.01	92.75	0.11	0.05	1.41	0.03
	St.Dev.	0.06	0.01	1.14	0.00	1.48	0.02	0.05	0.71	0.02
RK17 A00911	Average	0.19	bd	5.71	0.04	93.35	0.04	0.02	0.63	0.02
	St.Dev.	0.12	bd	1.57	0.01	1.69	0.00	0.01	0.13	0.01
RK17 A00912	Average	0.05	bd	7.32	0.03	91.37	0.06	bd	1.10	0.07
	St.Dev.	0.01	bd	2.08	0.00	2.01	0.00	bd	0.08	0.00
RK17 A00915	Average	0.52	bd	6.14	0.02	92.36	0.07	0.48	0.40	0.01
	St.Dev.	0.93	bd	1.90	0.01	2.79	0.00	0.19	0.06	0.00
RK17 A00951	Average	0.21	bd	1.99	0.01	97.49	0.22	0.02	0.05	bd
	St.Dev.	0.05	bd	0.50	0.00	0.45	0.03	0.00	0.02	bd
RK17 A00952	Average	0.60	bd	3.78	0.01	95.35	0.10	0.02	0.11	0.03
	St.Dev.	0.15	bd	0.85	0.00	0.90	0.00	0.01	0.04	0.02
RK17 A00953	Average	0.27	bd	4.13	0.01	95.31	0.11	0.08	0.08	bd
	St.Dev.	0.17	bd	1.51	0.00	1.38	0.01	0.02	0.01	bd
RK17 A00957	Average	0.22	bd	3.06	0.01	96.52	0.11	0.02	0.05	bd
	St.Dev.	0.23	bd	0.34	0.00	0.37	0.02	0.01	0.01	bd
RK17 A00958	Average	0.56	bd	1.64	0.01	97.43	0.29	0.03	0.03	bd
	St.Dev.	0.49	bd	0.54	0.00	0.91	0.04	0.03	0.01	bd
RK17 A00959	Average	0.50	bd	3.85	0.01	95.01	0.16	0.02	0.39	0.06
	St.Dev.	0.05	bd	0.18	0.00	0.27	0.01	0.00	0.09	0.00
RK17 A00960	Average	0.78	bd	2.84	0.01	95.98	0.25	0.04	0.04	0.04
	St.Dev.	0.79	bd	0.44	0.00	0.54	0.02	0.01	0.02	0.02
RK17 A00963	Average	0.29	bd	2.46	0.01	96.99	0.17	0.02	0.06	bd
	St.Dev.	0.02	bd	0.22	0.00	0.22	0.01	0.00	0.01	bd

Small find number		Fe	Ni	Cu	Zn	Ag	Au	Hg	Pb	Bi
RK17 A00972	Average	0.26	bd	2.57	0.01	97.05	bd	0.07	0.03	0.01
	St.Dev.	0.25	bd	1.74	0.00	1.50	bd	0.02	0.01	0.01
RK17 A00993	Average	bd	bd	4.06	0.01	94.64	0.04	0.05	1.13	0.07
	St.Dev.	bd	bd	1.03	0.00	1.00	0.00	0.01	0.05	0.00
RK17 A01023	Average	0.03	bd	5.93	0.01	93.83	bd	0.03	0.18	bd
	St.Dev.	0.03	bd	0.36	0.00	0.37	bd	0.00	0.01	bd
RK17 A01049	Average	0.06	bd	3.16	0.01	96.34	0.06	bd	0.32	0.05
	St.Dev.	0.04	bd	0.42	0.00	0.37	0.02	bd	0.02	0.01
RK17 A01050	Average	0.09	bd	2.58	0.03	97.10	bd	0.04	0.15	0.01
	St.Dev.	0.00	bd	0.64	0.00	0.66	bd	0.01	0.03	0.01
RK17 A01062	Average	0.43	bd	2.28	0.01	96.89	0.16	bd	0.21	0.03
	St.Dev.	0.48	bd	0.73	0.00	0.69	0.09	bd	0.02	0.01
RK17 A01077	Average	0.09	bd	5.73	0.01	92.96	0.02	0.06	1.01	0.11
	St.Dev.	0.06	bd	1.13	0.00	1.08	0.00	0.01	0.02	0.00
RK17 A01079	Average	0.02	bd	3.02	bd	96.67	0.12	0.02	0.15	0.01
	St.Dev.	0.01	bd	0.38	bd	0.40	0.01	0.01	0.04	0.01
RK17 A01082	Average	0.50	bd	7.14	0.02	92.04	0.09	bd	0.19	0.02
	St.Dev.	0.16	bd	0.75	0.00	0.90	0.00	bd	0.05	0.01
RK17 A01089	Average	0.78	bd	1.27	0.01	97.77	0.11	0.05	bd	0.01
	St.Dev.	0.67	bd	0.51	0.00	1.19	0.02	0.01	bd	0.00
RK17 A01092	Average	0.06	bd	1.91	bd	97.77	0.17	0.02	0.02	0.05
	St.Dev.	0.04	bd	0.11	bd	0.13	0.03	0.01	0.01	0.02
RK17 A01093	Average	0.20	bd	4.90	0.01	94.52	0.17	0.04	0.13	0.03
	St.Dev.	0.27	bd	1.26	0.00	0.96	0.01	0.06	0.05	0.00
RK17 A01099	Average	0.05	bd	3.09	bd	96.68	0.08	0.02	0.05	0.01
	St.Dev.	0.02	bd	0.37	bd	0.37	0.01	0.00	0.02	0.01
RK17 A01101	Average	bd	bd	3.04	0.01	96.69	0.17	bd	0.04	0.04
	St.Dev.	bd	bd	0.50	0.00	0.50	0.01	bd	0.01	0.01
RK17 A01107	Average	0.11	bd	3.91	0.01	95.74	0.13	bd	0.07	0.03
	St.Dev.	0.07	bd	1.07	0.00	1.13	0.01	bd	0.01	0.00
RK17 A01109	Average	1.15	bd	2.62	0.01	95.94	0.23	0.02	0.03	0.01
	St.Dev.	0.64	bd	0.68	0.00	0.30	0.03	0.01	0.01	0.00
RK17 A01111	Average	1.11	bd	1.64	0.01	97.04	0.13	0.03	0.03	0.01
	St.Dev.	0.44	bd	0.08	0.00	0.52	0.01	0.01	0.01	0.00
RK17 A01112	Average	1.09	bd	1.60	0.01	97.09	0.16	0.02	0.03	0.01
	St.Dev.	0.30	bd	0.10	0.00	0.34	0.01	0.00	0.01	0.00
RK17 A01114	Average	0.94	bd	1.93	0.01	96.86	0.16	0.02	0.04	0.05
	St.Dev.	0.49	bd	0.28	0.00	0.52	0.01	0.00	0.01	0.00
RK17 A01156	Average	bd	bd	4.19	0.01	95.49	bd	0.03	0.29	0.01
	St.Dev.	bd	bd	0.72	0.00	0.72	bd	0.01	0.01	0.00
RK17 A01182	Average	0.04	bd	0.45	bd	99.30	0.12	0.03	0.03	0.03
	St.Dev.	0.04	bd	0.61	bd	0.57	0.05	0.01	0.03	0.01
RK17 A01184	Average	bd	bd	2.11	bd	97.63	0.07	0.02	0.05	0.12
	St.Dev.	bd	bd	0.41	bd	0.42	0.01	0.01	0.01	0.01
RK17 A01187	Average	0.03	bd	4.91	0.01	94.71	0.10	0.02	0.20	0.02
	St.Dev.	0.03	bd	2.36	0.00	2.35	0.03	0.00	0.04	0.01
RK17 A01189	Average	0.16	bd	1.46	0.01	98.03	0.09	0.02	0.12	0.12
	St.Dev.	0.07	bd	0.68	0.00	0.74	0.02	0.01	0.03	0.01
RK17 A01190	Average	bd	bd	2.72	bd	97.05	0.11	bd	0.08	0.03
	St.Dev.	bd	bd	0.39	bd	0.38	0.00	bd	0.01	0.01
RK17 A01204	Average	0.50	bd	0.68	0.01	98.63	0.09	0.03	0.03	0.04
	St.Dev.	0.27	bd	0.20	0.00	0.23	0.01	0.00	0.00	0.01
RK17 A01208	Average	0.02	bd	3.06	0.01	96.70	0.08	0.02	0.12	bd

Small find number		Fe	Ni	Cu	Zn	Ag	Au	Hg	Pb	Bi
	St.Dev.	0.02	bd	0.30	0.00	0.29	0.01	0.00	0.01	bd
RK17 A01214	Average	0.02	bd	2.84	bd	96.90	0.06	bd	0.16	0.01
	St.Dev.	0.00	bd	0.37	bd	0.37	0.00	bd	0.01	0.00
RK17 A01219	Average	bd	bd	3.81	0.01	95.90	0.10	bd	0.16	0.03
	St.Dev.	bd	bd	0.17	0.00	0.17	0.00	bd	0.00	0.00
RK17 A01239	Average	0.03	bd	4.83	0.03	94.77	bd	bd	0.30	0.04
	St.Dev.	0.01	bd	0.28	0.01	0.28	bd	bd	0.02	0.00
RK17 A01241	Average	bd	bd	1.32	0.01	98.50	0.11	0.04	0.02	0.01
	St.Dev.	bd	bd	0.05	0.00	0.05	0.04	0.01	0.01	0.01
RK17 A01249	Average	0.63	bd	2.26	0.01	96.89	0.03	0.03	0.15	bd
	St.Dev.	0.50	bd	0.61	0.00	0.60	0.01	0.01	0.08	bd
RK17 A01270	Average	0.26	bd	4.88	0.03	94.61	0.02	0.02	0.18	bd
	St.Dev.	0.20	bd	0.57	0.00	0.40	0.01	0.01	0.01	bd
RK17 A01286	Average	0.25	bd	4.15	0.03	95.44	bd	0.04	0.09	bd
	St.Dev.	0.12	bd	0.39	0.00	0.41	bd	0.01	0.00	bd
RK17 A01302	Average	bd	bd	4.89	0.01	94.93	bd	0.02	0.15	bd
	St.Dev.	bd	bd	0.24	0.00	0.25	bd	0.00	0.01	bd
RK17 A01304	Average	0.02	bd	4.65	0.04	95.11	bd	0.03	0.16	bd
	St.Dev.	0.03	bd	0.27	0.01	0.29	bd	0.01	0.02	bd
RK17 A01305	Average	0.02	bd	3.51	0.03	96.24	0.03	bd	0.16	0.01
	St.Dev.	0.02	bd	0.17	0.00	0.19	0.00	bd	0.00	0.00
RK17 A01313	Average	0.60	bd	1.65	0.01	97.43	0.27	0.03	0.02	bd
	St.Dev.	0.43	bd	0.72	0.00	0.50	0.02	0.01	0.00	bd
RK17 A01331	Average	0.04	bd	2.77	0.01	96.44	0.04	0.02	0.66	0.02
	St.Dev.	0.02	bd	0.02	0.00	0.06	0.00	0.00	0.01	0.00
RK17 A02618	Average	0.10	0.01	2.30	0.01	97.18	bd	0.02	0.27	0.11
	St.Dev.	0.10	0.00	0.89	0.00	0.87	bd	0.00	0.09	0.04
RK17 A02619	Average	0.02	0.01	3.15	0.01	96.25	bd	0.02	0.38	0.16
	St.Dev.	0.02	0.00	1.71	0.00	1.82	bd	0.02	0.13	0.06
RK17 A02625	Average	bd	bd	3.00	0.02	96.59	0.04	0.09	0.24	0.01
	St.Dev.	bd	bd	0.71	0.00	0.68	0.01	0.06	0.04	0.00
RK17 A02628	Average	0.20	bd	3.09	0.01	96.22	bd	bd	0.34	0.14
	St.Dev.	0.04	bd	0.46	0.00	0.62	bd	bd	0.10	0.06
RK17 A02631	Average	0.04	0.01	3.95	0.01	95.59	bd	bd	0.31	0.09
	St.Dev.	0.05	0.00	0.79	0.00	0.79	bd	bd	0.04	0.01
RK17 A02632	Average	0.02	0.01	3.47	0.01	95.93	bd	bd	0.41	0.16
	St.Dev.	0.01	0.00	1.02	0.00	1.05	bd	bd	0.07	0.05
RK17 A02635	Average	0.49	0.01	1.32	0.01	97.90	0.03	bd	0.14	0.10
	St.Dev.	0.33	0.00	0.14	0.00	0.20	0.01	bd	0.03	0.03
RK17 A02642	Average	0.10	0.01	1.40	bd	97.98	bd	bd	0.29	0.21
	St.Dev.	0.06	0.00	0.16	bd	0.16	bd	bd	0.04	0.03
RK17 A02645	Average	0.05	bd	3.12	0.01	96.37	bd	bd	0.35	0.10
	St.Dev.	0.02	bd	0.89	0.00	0.90	bd	bd	0.09	0.03
RK17 A02651	Average	0.27	0.01	1.72	0.01	97.53	bd	bd	0.31	0.16
	St.Dev.	0.17	0.00	0.44	0.00	0.34	bd	bd	0.04	0.02
RK17 A02652	Average	0.25	bd	1.40	0.01	98.02	bd	0.02	0.18	0.10
	St.Dev.	0.15	bd	0.19	0.00	0.18	bd	0.01	0.06	0.04
RK17 A02653	Average	0.07	0.01	2.78	0.01	96.78	bd	bd	0.27	0.09
	St.Dev.	0.05	0.00	0.46	0.00	0.54	bd	bd	0.02	0.02
RK17 A02654	Average	0.04	0.01	2.27	0.01	97.32	bd	bd	0.24	0.12
	St.Dev.	0.02	0.00	0.24	0.00	0.30	bd	bd	0.07	0.05
RK17 A02655	Average	0.02	0.01	2.98	0.01	96.52	bd	bd	0.32	0.15
	St.Dev.	0.00	0.00	0.48	0.00	0.39	bd	bd	0.10	0.08

Small find number		Fe	Ni	Cu	Zn	Ag	Au	Hg	Pb	Bi
RK17 A02656 A	Average	0.04	bd	2.89	0.01	96.64	0.02	bd	0.32	0.08
	St.Dev.	0.04	bd	0.40	0.00	0.49	0.00	bd	0.17	0.06
RK17 A02656 B	Average	0.09	bd	3.02	0.01	96.43	bd	bd	0.27	0.16
	St.Dev.	0.10	bd	0.53	0.00	0.64	bd	bd	0.04	0.02
RK17 A02657	Average	0.05	bd	2.31	0.01	97.22	bd	bd	0.27	0.14
	St.Dev.	0.01	bd	0.36	0.00	0.38	bd	bd	0.07	0.00
RK17 A02658 B	Average	0.02	bd	2.58	0.01	97.08	bd	bd	0.22	0.08
	St.Dev.	0.02	bd	0.24	0.00	0.32	bd	bd	0.07	0.06
RK17 A02659	Average	0.42	0.01	1.72	0.01	97.60	0.02	0.02	0.15	0.06
	St.Dev.	0.36	0.00	0.26	0.00	0.11	0.00	0.01	0.08	0.02
RK17 A02660	Average	0.08	0.01	2.28	0.01	97.12	bd	0.03	0.31	0.17
	St.Dev.	0.02	0.00	0.29	0.00	0.33	bd	0.02	0.06	0.04
RK17 A02662	Average	0.33	0.01	1.87	0.01	97.20	bd	0.02	0.38	0.18
	St.Dev.	0.18	0.00	0.59	0.00	0.37	bd	0.01	0.04	0.03
RK17 A02669	Average	0.19	0.01	1.84	0.01	97.74	0.02	0.04	0.11	0.06
	St.Dev.	0.15	0.00	0.48	0.00	0.34	0.01	0.03	0.07	0.01
RK17 A02671	Average	0.29	0.01	1.94	bd	97.27	bd	0.03	0.29	0.17
	St.Dev.	0.25	0.00	0.60	bd	0.63	bd	0.03	0.19	0.11
RK17 A02694	Average	0.27	bd	2.77	0.01	96.71	bd	0.02	0.15	0.07
	St.Dev.	0.13	bd	0.33	0.00	0.38	bd	0.01	0.05	0.02
RK17 A02700	Average	0.08	bd	3.15	0.01	96.24	0.02	bd	0.38	0.12
	St.Dev.	0.07	bd	0.55	0.00	0.45	0.01	bd	0.04	0.02
RK17 A02701	Average	bd	0.01	5.63	0.01	93.53	bd	bd	0.64	0.18
	St.Dev.	bd	0.00	0.24	0.00	0.24	bd	bd	0.00	0.01
RK17 A02713	Average	0.19	0.01	3.70	0.01	95.64	bd	bd	0.31	0.14
	St.Dev.	0.12	0.00	0.87	0.00	0.85	bd	bd	0.04	0.06
RK17 A02724	Average	0.06	bd	3.81	0.01	95.77	0.02	bd	0.26	0.07
	St.Dev.	0.04	bd	0.60	0.00	0.59	0.00	bd	0.07	0.03
RK17 A02726	Average	0.11	0.01	2.44	0.01	97.14	0.02	bd	0.22	0.06
	St.Dev.	0.08	0.01	0.77	0.02	0.82	0.00	bd	0.02	0.01
RK17 A02727	Average	0.08	0.01	4.11	0.01	95.38	bd	bd	0.31	0.10
	St.Dev.	0.01	0.00	1.69	0.00	1.79	bd	bd	0.11	0.02
RK17 A02729	Average	0.84	0.01	1.89	0.01	96.80	0.03	0.02	0.27	0.14
	St.Dev.	0.45	0.00	0.68	0.00	0.99	0.01	0.01	0.05	0.06
RK17 A02733	Average	0.18	bd	3.14	0.01	96.38	0.02	0.04	0.23	0.01
	St.Dev.	0.15	bd	0.94	0.00	0.85	0.01	0.02	0.06	0.00
RK17 A02740	Average	0.19	bd	2.19	0.01	97.26	0.02	bd	0.22	0.11
	St.Dev.	0.17	bd	0.92	0.00	0.76	0.01	bd	0.02	0.01
RK17 A02741	Average	0.09	0.01	2.76	0.01	96.69	bd	bd	0.31	0.13
	St.Dev.	0.07	0.00	0.58	0.00	0.64	bd	bd	0.06	0.06
RK17 A02750	Average	3.02	0.01	1.61	0.01	94.96	0.02	0.04	0.20	0.12
	St.Dev.	2.43	0.00	0.31	0.00	1.98	0.01	0.02	0.08	0.06
RK17 A02755	Average	0.20	bd	0.28	bd	99.45	bd	0.04	0.02	0.01
	St.Dev.	0.16	bd	0.14	bd	0.29	bd	0.00	0.00	0.00
RK17 A02760	Average	0.31	0.01	2.45	0.01	96.88	0.02	0.03	0.22	0.07
	St.Dev.	0.31	0.00	0.89	0.00	0.71	0.01	0.02	0.02	0.03
RK17 A02771	Average	0.05	bd	3.26	0.03	95.93	0.06	bd	0.61	0.05
	St.Dev.	0.02	bd	0.39	0.02	0.50	0.00	bd	0.15	0.01
RK17 A02775	Average	0.28	0.01	3.29	0.01	95.89	bd	bd	0.37	0.14
	St.Dev.	0.36	0.00	0.81	0.00	0.74	bd	bd	0.14	0.07
RK17 A02780	Average	0.16	bd	3.94	0.01	95.66	0.03	bd	0.18	bd
	St.Dev.	0.15	bd	0.24	0.00	0.22	0.00	bd	0.02	bd
RK17 A02785	Average	0.20	0.01	1.59	0.01	97.87	0.02	0.02	0.16	0.14

Small find number		Fe	Ni	Cu	Zn	Ag	Au	Hg	Pb	Bi
	St.Dev.	0.11	0.00	0.51	0.00	0.29	0.00	0.00	0.04	0.10
RK17 A02788	Average	0.19	bd	4.16	0.01	95.38	bd	0.02	0.23	bd
	St.Dev.	0.19	bd	0.80	0.00	0.64	bd	0.00	0.02	bd
RK17 A02798	Average	0.11	0.01	1.06	0.01	98.63	0.02	0.03	0.10	0.04
	St.Dev.	0.03	0.00	0.63	0.00	0.57	0.00	0.00	0.02	0.02
RK17 A02801	Average	0.02	0.01	3.13	0.01	96.40	bd	bd	0.32	0.11
	St.Dev.	0.01	0.00	0.42	0.00	0.44	bd	bd	0.06	0.05
RK17 A02819	Average	bd	bd	4.04	0.02	95.43	bd	bd	0.49	0.01
	St.Dev.	bd	bd	0.14	0.00	0.12	bd	bd	0.04	0.00
RK17 A02820	Average	bd	bd	3.17	0.01	96.71	bd	0.03	0.07	bd
	St.Dev.	bd	bd	0.87	0.00	0.85	bd	0.02	0.02	bd
RK17 A02821 B	Average	0.02	bd	4.94	0.01	94.65	0.02	bd	0.34	0.02
	St.Dev.	0.02	bd	0.32	0.00	0.35	0.00	bd	0.06	0.00
RK17 A02822	Average	0.15	bd	4.31	0.02	95.27	bd	0.02	0.24	bd
	St.Dev.	0.11	bd	0.39	0.00	0.29	bd	0.00	0.03	bd
RK17 A02823	Average	0.02	bd	4.57	0.01	95.22	bd	0.03	0.15	bd
	St.Dev.	0.01	bd	0.59	0.00	0.60	bd	0.00	0.02	bd
RK17 A02824 E	Average	0.04	bd	4.19	0.02	95.28	0.02	bd	0.44	bd
	St.Dev.	0.05	bd	0.32	0.01	0.36	0.00	bd	0.00	bd
RK17 A02825	Average	0.02	0.01	4.86	0.03	94.97	bd	0.02	0.10	bd
	St.Dev.	0.01	bd	0.21	0.00	0.20	bd	0.00	0.00	bd
RK17 A02826	Average	0.19	bd	4.35	0.03	95.16	bd	0.02	0.25	bd
	St.Dev.	0.36	bd	0.17	0.03	0.47	bd	0.02	0.02	bd
RK17 A02827	Average	bd	bd	4.33	0.02	95.14	0.04	bd	0.45	bd
	St.Dev.	bd	bd	0.28	0.00	0.29	0.00	bd	0.02	bd
RK17 A02829	Average	bd	bd	3.95	0.02	95.94	bd	0.03	0.07	bd
	St.Dev.	bd	bd	0.57	0.00	0.58	bd	0.00	0.01	bd
RK17 A02831	Average	bd	0.01	0.61	0.01	99.22	bd	0.05	0.11	bd
	St.Dev.	bd	0.00	0.33	0.01	0.38	bd	0.02	0.08	bd
RK17 A02840	Average	bd	bd	5.26	0.02	94.30	0.02	bd	0.39	0.01
	St.Dev.	bd	bd	0.32	0.00	0.32	0.00	bd	0.01	0.00
RK17 A02842	Average	bd	bd	4.98	0.01	94.74	bd	0.02	0.24	bd
	St.Dev.	bd	bd	0.24	0.00	0.25	bd	0.01	0.01	bd
RK17 A02843	Average	0.06	bd	4.07	0.02	95.63	0.03	0.02	0.18	bd
	St.Dev.	0.03	bd	0.28	0.01	0.28	0.01	0.00	0.02	bd
RK17 A02844	Average	0.05	bd	3.88	0.02	95.74	0.06	0.04	0.22	bd
	St.Dev.	0.02	bd	0.24	0.00	0.22	0.01	0.01	0.01	bd
RK17 A02845	Average	0.08	bd	4.17	0.02	95.51	bd	0.02	0.19	bd
	St.Dev.	0.06	bd	0.28	0.01	0.30	bd	0.01	0.04	bd
RK17 A02846	Average	0.29	bd	4.93	0.02	94.59	bd	0.02	0.14	bd
	St.Dev.	0.29	bd	0.48	0.01	0.19	bd	0.01	0.03	bd
RK17 A02847	Average	0.03	bd	3.73	0.01	96.07	bd	0.03	0.12	bd
	St.Dev.	0.03	bd	0.29	0.00	0.29	bd	0.01	0.03	bd
RK17 A02849	Average	0.08	bd	3.59	0.01	95.88	bd	bd	0.44	bd
	St.Dev.	0.08	bd	0.82	0.00	0.78	bd	bd	0.10	bd
RK17 A02850	Average	0.02	bd	4.95	0.04	94.81	bd	bd	0.16	0.01
	St.Dev.	0.02	bd	0.30	0.00	0.29	bd	bd	0.02	0.01
RK17 A02851	Average	0.05	bd	4.48	0.02	95.16	0.03	bd	0.25	0.01
	St.Dev.	0.07	bd	0.23	0.00	0.25	0.00	bd	0.04	0.01
RK17 A02852	Average	0.04	bd	4.52	0.02	95.16	bd	bd	0.24	0.01
	St.Dev.	0.03	bd	0.21	0.01	0.22	bd	bd	0.01	0.00
RK17 A02853	Average	0.26	bd	3.57	0.02	95.72	0.02	0.02	0.24	0.15
	St.Dev.	0.29	bd	0.32	0.00	0.22	0.01	0.01	0.03	0.03

Small find number		Fe	Ni	Cu	Zn	Ag	Au	Hg	Pb	Bi
RK17 A02859 A	Average	0.10	bd	3.49	0.03	96.25	bd	0.02	0.10	bd
	St.Dev.	0.06	bd	0.64	0.00	0.61	bd	0.01	0.01	bd
RK17 A02860 C	Average	bd	bd	4.08	0.03	95.66	0.13	0.02	0.07	0.01
	St.Dev.	bd	bd	0.20	0.00	0.22	0.01	0.00	0.02	0.00
RK17 A02860 D	Average	bd	bd	2.95	0.01	96.15	0.07	bd	0.70	0.12
	St.Dev.	bd	bd	0.48	0.00	0.80	0.00	bd	0.27	0.07
RK17 A02861	Average	0.02	bd	3.51	0.01	96.31	0.02	0.02	0.09	0.02
	St.Dev.	0.03	bd	1.04	0.00	1.01	0.01	0.01	0.01	0.01
RK17 A02862	Average	0.06	bd	4.72	0.02	94.97	0.02	bd	0.19	0.02
	St.Dev.	0.09	bd	0.68	0.01	0.64	0.00	bd	0.04	0.01
RK17 A02864	Average	0.05	bd	4.13	0.02	95.36	0.08	bd	0.35	0.01
	St.Dev.	0.04	bd	0.40	0.00	0.43	0.01	bd	0.10	0.01
RK17 A02865	Average	0.02	bd	4.39	0.01	95.18	0.03	bd	0.34	0.04
	St.Dev.	0.01	bd	0.15	0.00	0.14	0.00	bd	0.03	0.00
RK17 A02872	Average	0.07	0.01	4.61	0.02	95.09	bd	0.02	0.18	bd
	St.Dev.	0.09	0.00	0.26	0.01	0.21	bd	0.00	0.01	bd
RK17 A02874	Average	0.03	bd	3.39	0.03	95.94	0.03	0.11	0.48	bd
	St.Dev.	0.02	bd	0.04	0.01	0.01	0.00	0.01	0.03	bd
RK17 A02875	Average	0.03	bd	4.97	0.02	93.55	0.06	0.79	0.58	0.01
	St.Dev.	0.01	bd	0.17	0.00	0.87	0.01	0.46	0.04	0.00
RK17 A02878	Average	0.03	bd	3.78	0.03	94.67	0.06	0.43	1.01	bd
	St.Dev.	0.03	bd	0.58	0.01	0.55	0.01	0.10	0.10	bd
RK17 A02893	Average	bd	bd	10.86	0.03	88.77	0.03	0.02	0.30	bd
	St.Dev.	bd	bd	4.26	0.01	4.24	0.00	0.01	0.08	bd
RK17 A02896	Average	bd	bd	5.63	0.03	93.36	0.07	0.34	0.55	0.01
	St.Dev.	bd	bd	1.45	0.00	1.30	0.00	0.08	0.14	0.00
RK17 A02897	Average	1.91	bd	21.82	0.07	75.47	0.09	bd	0.62	0.03
	St.Dev.	0.48	bd	3.37	0.01	3.33	0.00	bd	0.03	0.00
RK17 A02898	Average	0.71	0.01	4.38	0.06	94.57	0.02	0.02	0.24	bd
	St.Dev.	0.57	0.00	0.32	0.03	0.78	0.00	0.00	0.09	bd
RK17 A02901	Average	0.07	bd	5.31	0.03	94.00	0.02	0.02	0.54	0.02
	St.Dev.	0.04	bd	1.30	0.01	1.27	0.00	0.00	0.02	0.00
RK17 A02908	Average	0.64	bd	3.84	0.03	95.10	0.02	bd	0.37	0.01
	St.Dev.	0.16	bd	0.43	0.00	0.55	0.00	bd	0.02	0.00
RK17 A02909	Average	0.04	bd	21.45	0.03	77.52	0.02	bd	0.90	0.04
	St.Dev.	0.01	bd	3.25	0.00	3.36	0.00	bd	0.19	0.01
RK17 A02910	Average	0.02	bd	4.41	0.02	95.04	0.04	0.02	0.44	0.01
	St.Dev.	0.01	bd	0.84	0.00	0.78	0.00	0.00	0.02	0.00
RK17 A02911	Average	0.14	bd	5.10	0.05	93.99	bd	0.06	0.66	0.01
	St.Dev.	0.05	bd	0.84	0.01	0.86	bd	0.02	0.05	0.00
RK17 A02915	Average	0.02	bd	4.83	0.03	94.05	0.03	0.61	0.42	0.01
	St.Dev.	0.01	bd	1.20	0.00	1.12	0.00	0.36	0.07	0.00
RK17 A02917	Average	0.02	bd	4.61	0.02	94.05	0.04	0.02	1.09	0.14
	St.Dev.	0.02	bd	0.46	0.01	0.63	0.00	0.02	0.27	0.03
RK17 A02922	Average	0.12	0.05	28.72	0.23	70.07	0.07	bd	0.74	0.01
	St.Dev.	0.05	0.01	1.88	0.04	1.80	0.01	bd	0.38	0.01
RK17 A02933	Average	0.04	bd	3.51	0.03	95.89	bd	0.07	0.46	0.01
	St.Dev.	0.02	bd	0.17	0.00	0.20	bd	0.03	0.06	0.00
RK17 A02935	Average	0.03	bd	3.55	0.02	95.83	0.02	0.03	0.50	0.03
	St.Dev.	0.01	bd	0.15	0.00	0.16	0.00	0.01	0.03	0.00
RK17 A02936	Average	0.04	bd	8.46	0.03	90.79	0.05	0.04	0.56	0.04
	St.Dev.	0.00	bd	1.23	0.00	1.20	0.00	0.00	0.03	0.00
RK17 A02937	Average	0.35	bd	3.66	0.04	95.65	0.02	0.04	0.24	bd

Small find number		Fe	Ni	Cu	Zn	Ag	Au	Hg	Pb	Bi
	St.Dev.	0.21	bd	0.87	0.01	1.18	0.00	0.01	0.15	bd
RK17 A02938	Average	0.65	bd	5.85	0.02	93.04	0.03	0.07	0.33	0.01
	St.Dev.	0.90	bd	1.49	0.01	0.91	0.00	0.07	0.17	0.00
RK17 A02942	Average	0.02	bd	3.80	0.01	95.49	0.05	0.10	0.52	0.02
	St.Dev.	0.01	bd	0.33	0.00	0.40	0.00	0.04	0.08	0.00
RK17 A02947	Average	0.03	bd	3.64	0.01	95.59	0.06	0.06	0.61	0.01
	St.Dev.	0.01	bd	0.57	0.00	0.59	0.00	0.01	0.07	0.00
RK17 A02959	Average	bd	bd	4.36	0.01	95.36	bd	bd	0.26	bd
	St.Dev.	bd	bd	0.34	0.00	0.39	bd	bd	0.05	bd
RK18 A00128	Average	bd	bd	2.15	0.01	97.66	0.06	0.11	bd	0.02
	St.Dev.	bd	bd	0.43	0.00	0.41	0.01	0.02	bd	0.01
RK18 A00129	Average	0.14	bd	2.85	0.01	96.80	0.06	0.02	0.09	0.03
	St.Dev.	0.18	bd	1.33	0.00	1.19	0.02	0.01	0.04	0.01
RK18 A00230	Average	bd	bd	4.41	0.02	94.93	0.03	0.08	0.51	0.01
	St.Dev.	bd	bd	0.21	0.00	0.26	0.00	0.04	0.11	0.01
RK18 A00239	Average	0.08	bd	4.09	0.01	95.58	bd	0.02	0.22	bd
	St.Dev.	0.12	bd	0.19	0.00	0.10	bd	0.00	0.01	bd
RK18 A00241	Average	0.96	bd	0.90	0.01	97.89	0.16	0.04	0.02	0.03
	St.Dev.	0.57	bd	0.46	0.00	1.00	0.02	0.00	0.01	0.00
RK18 A00259	Average	0.81	bd	8.83	0.04	88.85	0.03	0.78	0.66	0.01
	St.Dev.	0.06	bd	1.83	0.01	2.22	0.00	0.36	0.02	0.00
RK18 A00268	Average	bd	bd	4.26	0.02	95.52	bd	0.02	0.18	bd
	St.Dev.	bd	bd	0.46	0.00	0.48	bd	0.00	0.01	bd
